# The role of m^6^A, m^5^C and Ψ RNA modifications in cancer: Novel therapeutic opportunities

**DOI:** 10.1186/s12943-020-01263-w

**Published:** 2021-01-18

**Authors:** Paz Nombela, Borja Miguel-López, Sandra Blanco

**Affiliations:** 1 Centro de Investigación del Cáncer and Instituto de Biología Molecular y Celular del Cáncer, Consejo Superior de Investigaciones Científicas (CSIC) - University of Salamanca, 37007 Salamanca, Spain; 2 Instituto de Investigación Biomédica de Salamanca (IBSAL), Hospital Universitario de Salamanca, 37007 Salamanca, Spain

**Keywords:** Cancer, Inhibitors, Anti-cancer therapy, Proliferation, Migration, Epitranscriptome, RNA modifications, N6-methyladenosine, m^6^A, 5-methylcytosine, m^5^C, Pseudouridine, Ψ

## Abstract

RNA modifications have recently emerged as critical posttranscriptional regulators of gene expression programmes. Significant advances have been made in understanding the functional role of RNA modifications in regulating coding and non-coding RNA processing and function, which in turn thoroughly shape distinct gene expression programmes. They affect diverse biological processes, and the correct deposition of many of these modifications is required for normal development. Alterations of their deposition are implicated in several diseases, including cancer. In this Review, we focus on the occurrence of N^6^-methyladenosine (m^6^A), 5-methylcytosine (m^5^C) and pseudouridine (Ψ) in coding and non-coding RNAs and describe their physiopathological role in cancer. We will highlight the latest insights into the mechanisms of how these posttranscriptional modifications influence tumour development, maintenance, and progression. Finally, we will summarize the latest advances on the development of small molecule inhibitors that target specific writers or erasers to rewind the epitranscriptome of a cancer cell and their therapeutic potential.

## Introduction

The epitranscriptome landscape is very complex, with more than 170 different types of chemical modifications of RNA described to date to decorate coding and non-coding RNAs (ncRNAs) [1]. Their occurrence has been well documented for over 50 years, however their function remains still widely unknown [2]. Thus, while known since the emergence of molecular biology, RNA modifications were only coined as the “epitranscriptome” in 2015. The study of the function of these modifications is now emerging and has shown to have big implications in human pathologies [3, 4]. For example, the role of 6-methyladenosine (m^6^A), the most abundant and better characterized internal modification in messenger RNA (mRNA), is to regulate embryonic stem cells and cancer cells self-renewal and to favour survival upon heat shock or DNA damage [5–7]. In addition to the roles of m^6^A modification in mRNAs, adenosine methylation is also found in non-coding RNAs, such as microRNAs (miRNAs), long non-coding RNAs (lncRNAs), and circular RNAs (circRNAs) regulating their biogenesis and function [8–15]. We now begin to appreciate the plethora of molecular processes that are finely regulated by RNA modifications ranging from RNA metabolism, decay, splicing or translation, localization, stability, turnover, binding to RNA binding proteins (RBPs) or other RNAs, and thereby diversifying genetic information. Similar to epigenetics, groups of proteins have been identified that specifically “write” (catalyse the deposition of a specific modification), “erase” (catalyse the removal of a specific modification), and “read” (recognize and bind modified nucleotides) thereby affecting the fate of RNA. Other modifications have been recently documented in mRNA including N6,2′-O-dimethyladenosine (m^6^Am), 5-methylcytosine (m^5^C), 5-hydroxymethylcytosine (hm^5^C), pseudouridine (Ψ), 1-methyladenosine (m^1^A) or 2'-O-ribose methylation, although their molecular functions remain still widely unknown [5, 16].

RNA modifications are also present in other regulatory ncRNAs, in fact the most modified RNAs are transfer RNA (tRNAs) and ribosomal RNAs (rRNAs) and their modifications shape protein synthesis efficiency and fidelity. More than 100 modifications have been described for tRNA, being the anticodon loop one hotspot of modifications and play key roles in accurate and efficient decoding in translation [17]. In rRNA, most modifications cluster around functional sites including the decoding site and the peptidyl transfer centre (PTC), suggesting their functional relevance in regulating protein synthesis [18, 19]. Studies in humans, yeast, and bacteria have shown that dynamic deposition of these modifications in rRNA regulate cell growth, and drug and stress sensitivity by fine-tuning translation and is a very conserved mechanism [17, 20–22]. For instance, in yeast, flies, worms, and humans, alterations of m^5^C levels in rRNA favours the translation of stress response-decoding transcripts in order to increase survival [23, 24]. Occurrence of Ψ residues increases in mRNA in yeast under starvation and heat shock [25–27]. And lack of 2′-O-ribose methylations in rRNAs decreases efficient translation and affects growth and sensitivity to antibiotics [28]. Similarly, the overall levels of tRNA modifications change to reprogram protein translation by changing codon usage [29–31].

The deposition of RNA modifications is dynamic, and thereby allows rapid cellular responses to environmental signals [16, 25, 31–34]. The ability to adapt to changing microenvironments such as that of stress or chemotherapeutic drugs is crucial to ensure survival of tumour cells, indicating that RNA modifications could play important roles in cancer. Historically, cancer has been considered fundamentally as a disease characterized by stepwise accumulation of genetic or epigenetic alterations of different oncogenes and tumour suppressor genes. However, compelling evidence indicates that epitranscriptomics could also play a fundamental role in this pathology. Through its ability to modulate many processes of RNA metabolism, dynamic RNA modifications have been shown to be important emerging regulators in cancer [3, 33, 35–38]. Although RNA modifications are not generally considered cancer drivers, cumulative evidence shows that their aberrant expression is functionally related to survival, proliferation, self-renewal, differentiation, stress adaptation, invasion, and resistance to therapy, all of which are hallmarks of cancer [24, 33, 35, 37, 39–43]. For example, dynamic changes for multiple RNA modifications can be observed in the urine of cancer patients [44]. Most striking it has been the extraordinary enlargement of experimental evidence that implicates alterations in the expression of m^6^A writers, erasers or readers are associated with increased risk of obesity and diabetes, infertility and with tumour-suppressive or tumour-promoting scenarios [3, 45]. Other RNA modifying enzymes have been found to be altered in cancer. For example, in an aggressive breast cancer cell lines, 2′-O methylation appeared to be hypermodified in rRNA and correlated with altered translation [46]. Mutations in the rRNA pseudouridine synthase DKC1, cause X-linked dyskeratosis congenita (X-DC) characterized by impaired translation, hematopoietic stem cells differentiation failure and increased cancer susceptibility [47]. Alterations in tRNA modifications have been also reported in cancer including m^5^C or 5-methoxycarbonylmethyluridine (mcm^5^U) and correlate with altered protein translation [33, 48–51]. All these studies show that aberrant RNA modifications contribute to proliferation, self-renewal, migration, stress adaptation and survival of cancer cells and suggest that targeting aberrant posttranscriptional modifications in cancer cells may hold promise as an efficient therapy for tumours [52].

In this review we will discuss the molecular and cellular functions of RNA modifications in modulating gene expression programmes, with a focus on their roles in cancer. We further summarize here recent studies that elucidate the therapeutic potential of targeting their aberrant deposition in cancer. We will focus our review article on m^6^A, m^5^C and Ψ in coding and non-coding RNAs as notable examples due to the advances in our understanding of the role of these epitranscriptomic marks in cellular functions including proliferation, self-renewal, survival to stress or migration. In addition, expression alterations or mutations in m^6^A, m^5^C and Ψ depositing machineries have been documented in cancer.

## 6-methyladenosine

### m^6^A deposition in coding and non-coding RNA

**Fig. 1:**
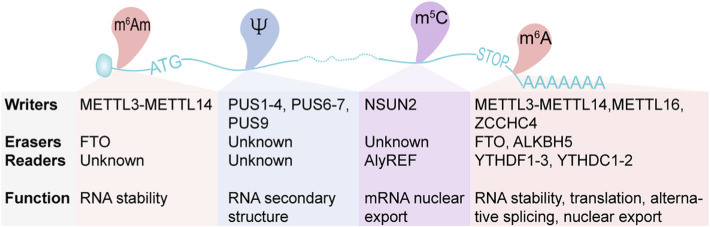
Schematic diagram of the location of m^6^A, m^6^Am, m^5^C and Ψ modifications on mRNA. Blue ribbon represents the mRNA with m^7^G-cap and a poly(A) tail. ATG and STOP codons are indicated. The writers, erasers, readers and function are listed in the text box attached to each modification. Modifications: m^6^A, 6-methyladenosine; m^6^Am, N^6^,2ʹ-O-dimethyladenosine; m^5^C, 5-methylcytosine; Ψ, pseudouridine. Proteins: METTL, methyltransferase-like; FTO, fat mass and obesity-associated protein; PUS, pseudouridine synthase; NSUN, NOL1/NOP2/SUN domain family member; ALyREF, Aly/REF Export Factor; ZCCHC4, zinc-finger CCHC domain-containing protein 4; ALKBH5, Alpha-Ketoglutarate-Dependent Dioxygenase AlkB Homolog 5; YTHDC, YTH domain-containing; YTHDF, YTH domain-containing family

N6-methyladenosine (m^6^A), a well-known posttranscriptional modification first discovered in 1974 [53, 54], has been regarded as the most frequent internal modification found in mRNA from viruses to mammals, but also occurs in small ncRNA and lncRNA in many eukaryotic species [11, 55]. Around 0.1–0.4% of all mRNA adenines are methylated at position N^6^, representing approximately 3–5 modifications per mRNA (Fig. 1) [56, 57]. The recent advent of genome-wide m^6^A mapping of polyadenylated RNAs has yielded unprecedented insights into the m^6^A-methylome landscape. Most methods for global m^6^A detection rely on immunoprecipitation of methylated RNAs using specific antibodies that recognise m^6^A [32, 58]. Subsequent improvements using ultraviolet crosslinking steps to bind the methylated RNA to antibodies have allowed the identification of m^6^A sites at single-nucleotide resolution [59, 60]. These methods have revealed that this modification is enriched at 3′ untranslated regions (3′UTRs), near stop codons, within long internal exons, in intergenic regions, introns, and at 5’UTRs (Fig. 1) [32, 41, 59–61].

Deposition of m^6^A has been also reported in ncRNAs such as miRNA, lncRNA and circRNA [9, 14, 62–65]. Deposition in miRNA is enriched in primary miRNAs (pri-miRNA), but not in precursor miRNAs (pre-miRNA) and m^6^A marks are usually located in both intergenic and intragenic pri-miRNAs that contain canonical METTL3 motifs [9]. As for circRNAs, despite being derived from mRNA exons, m^6^A-modified circRNAs are frequently derived from exons not methylated in mRNAs [62]. LncRNAs are generally defined as transcripts longer than 200 nucleotides, and yet despite sharing several features with coding mRNAs such as being 5′capped, spliced and, polyadenylated, m^6^A residues in lncRNAs are distributed along the whole body of the transcript and are more present in lncRNAs that undergo alternative splicing [65].

#### m^6^A writers

**Fig. 2:**
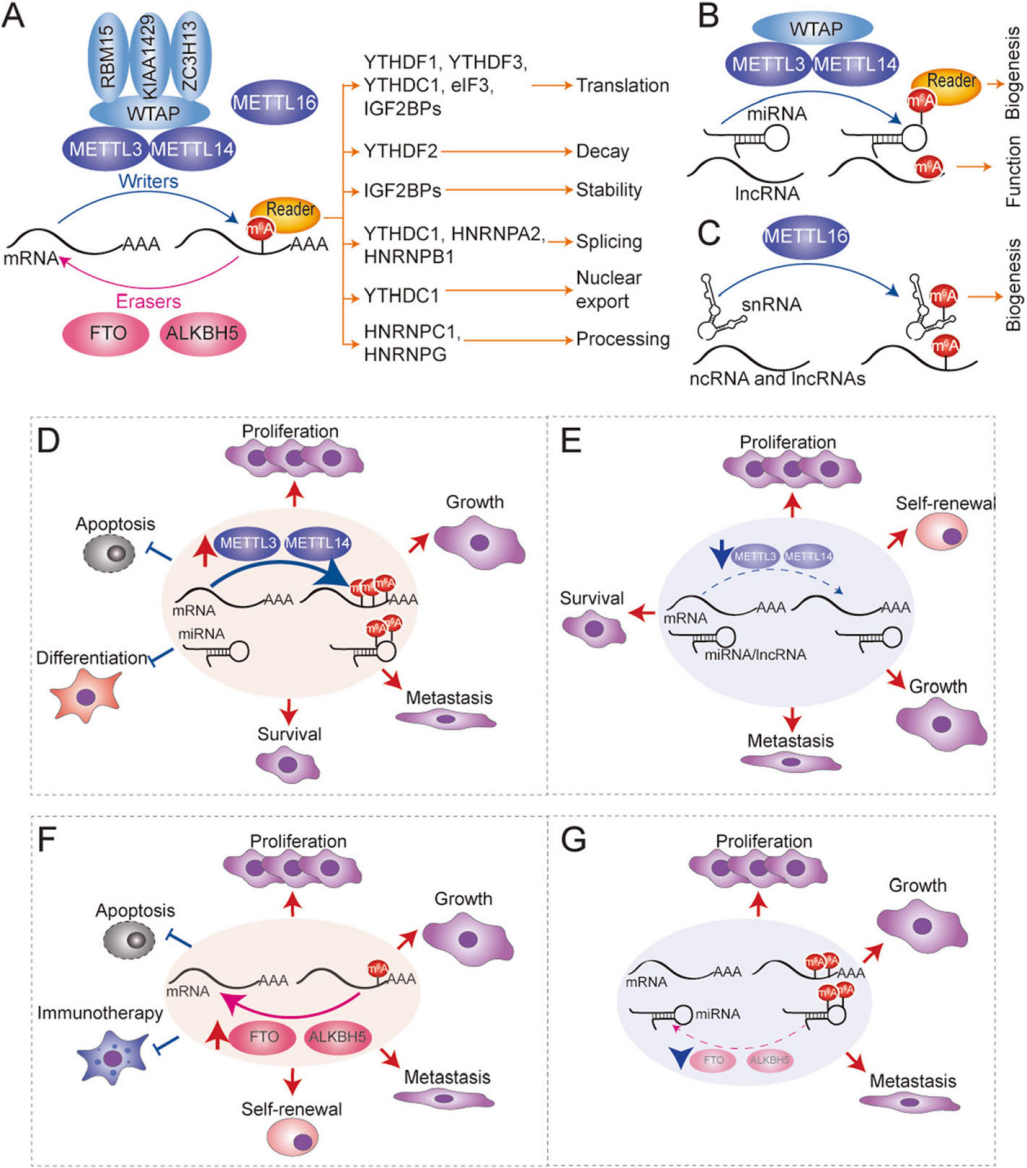
Molecular mechanism of m^6^A deposition in RNA, biological function and implications in human cancer. **a**-**c** m^6^A RNA methylation landscape in mRNA (**a**) and ncRNA (**b** & **c**) mediated by writers (blue balloons), including METTL3, METTL14, WTAP, RBM15B, KIAA1429, ZC3H13 and METTL16, erasers (pink balloons) FTO and ALKBH5 and reader proteins (yellow balloons) YTHDF1, YTHDF2, YTHDF3, YTHDC1, YTHDC2 and HNRNPC, HNRNPG, HNRNPA2, HNRNPB1, IGF2BPs and eIF3, and their role in mRNA metabolism. **d**-**g** Aberrant m^6^A deposition in mRNA and ncRNAs promotes or suppresses tumour progression through METTL3/METTL14 upregulation (**d**) or downregulation (**e**) and FTO/ALKBH5 upregulation (**f**) or downregulation (**g**). Red arrows indicate induction. Blue arrows with flat end represent inhibition

The deposition of m^6^A occurs into nascent pre-mRNAs during transcription and it is carried out in the nucleus by a multicomponent methyltransferase complex, that includes the S-adenosyl methionine (SAM) binding protein methyltransferase-like 3 (METTL3) and methyltransferase-like 14 (METTL14) heterodimeric catalytic core (Fig. 2a) [66–68]. METTL3 catalyses the conversion of adenosine to m^6^A through its methyltransferase domain, while METTL14 is responsible for the recognition of RNA substrates, and therefore the whole METTL3-METTL14 heterodimer is required for the methylation process [69, 70]. In addition, the currently defined methyltransferase complex is also composed of adapters. The RNA-binding motif protein 15 (RBM15) is one of these adapters and is responsible for the initial recruitment of the complex to its target site in the mRNA. The regulatory proteins Wilms’ tumour 1-associating protein (WTAP) and KIAA1429 (also known as VIRMA) are responsible for the complex formation [71, 72]. The recently characterized zinc finger CCCH domain-containing protein 13 (ZC3H13) has been found to act as a bridge between the adaptor RBM15 and WTAP [73]. miRNAs are methylated by the METTL3-METTL14-WTAP-RBM15/15B-KIAA1429 complex [9]. More recently, a new study has identified a single enzyme, METTL16, as another active m^6^A methyltransferase in human cells [74]. METTL16 has been shown to methylate mostly small nuclear RNAs, a number of intronic sites in pre-mRNAs and in addition other ncRNAs (Fig. 2c) [15, 74, 75].

#### m^6^A erasers

Deposition of N^6^-methyladenosine is reversible and relies on an orchestrated and dynamic network of specific methyltransferases but also demethylases or “erasers”. Two 6-methyladenosine demethylases have been identified, fat mass and obesity-associated protein (FTO) and AlkB homolog 5 (ALKBH5), which are members of the nonheme Fe (II)/α-ketoglutarate-dependent dioxygenases (Figs. 1 and 2a) [76, 77]. FTO shows a preference for demethylating N^6^,2′O-dimethyladenosine (m^6^Am) but also demethylates m^6^A [78, 79]. Furthermore, the AlkB homolog 3 (ALKBH3), another member of this family which preferentially acts on m^6^A in tRNAs [80]. While we start to appreciate the complexity of the m^6^A methylation machinery, yet it remains to be fully understood how the methylation machinery selectively and dynamically targets specific regions of the transcriptome.

#### m^6^A readers

m^6^A methylation acts as a unique recognition element for reader binding proteins that drive the biochemical processes that occurred to marked RNAs [81]. Some of the m^6^A readers contain a common RNA binding domain, the YTH domain, which include the family of YTH domain-containing proteins 1 and 2 (YTHDC1 and YTHDC2, respectively) and the YTH domain family proteins 1, 2 and 3 (YTHDF1, YTHDF2 and YTHDF3, respectively) [82, 83]. Subsequently, other readers have been discovered; eukaryotic translation initiation factor 3 (eIF3), heterogeneous nuclear ribonucleoprotein (HNRNPC and HNRNPA2/B1), insulin-like growth factor (IGF2BP1, IGF2BP2, and IGF2BP3), proline-rich and coiled-coil-containing protein 2A (PRRC2A), and the fragile X mental retardation protein FMRP, among other protein readers described to date (Figs. 1 and 2a) [8, 13, 61, 84–87].

### Molecular function of m^6^A deposition in RNA

#### Role of m^6^A in mRNA

Regarding the biological function of m^6^A deposition, this dependents on the protein reader that identifies and binds the modified mRNA (Fig. 2a). For example, the YTHDC protein subtypes are found mainly in the nucleus, and specifically YTHDC1 has been described as an alternative splicing factor of pre-mRNA [88]. YTHDC1 facilitates mRNA export too by recruiting nuclear transport receptors or by binding to m^6^A methyltransferases complexed with TREX mRNA export complex and modified mRNAs [89, 90]. In contrast, YTHDF protein subtypes are predominantly found in the cytoplasm and regulate the fate of cytoplasmic modified mRNAs. For example, YTHDF1 and YTHDF3 improve the efficiency of mRNA translation [91, 92], and YTHDF2 facilitates mRNA decay through the CC4-NOT deadenylase complex [93, 94]. YTHDF3 may accelerate mRNA decay too through interacting with YTHDF2 [92]. YTHDC2 protein can exist both inside and outside the nucleus, being able to selectively bind to m^6^A mark of ncRNA, yet the biological consequence upon binding is unknown [11]. In the cytosol, YTHDC2 affects the translation efficiency and abundance of its target mRNAs [95]. While the evidence so far has shown that m^6^A deposition mediate diverse effects through a complex network of readers, a recent study has revealed evidence for a unifying functional model of m^6^A readers, where m^6^A predominantly influences mRNA degradation through the combined action of three YTHDF member readers [96].

Apart from the YTHDC and YTHDF members, other reader proteins can modulate translation or stability. For instance, eIF3 subunits have been reported to affect canonical and non-canonical cap-dependent translation. This protein binds preferentially to m^6^A within the 5′ UTR of mRNA leading to enhanced translation [61]. In addition, evidence shows that IGF2BP promotes the stability and storage of their targeted mRNAs [87].

It must be considered too that the methylation of adenines causes alterations in the secondary structure of RNAs, which in turn may alter their interaction with reader proteins [97]. m^6^A compared to A promotes the destabilization of A/U pairings and alters the thermostability of RNA duplexes [98]. This structural change has been already correlated with an alteration in the interaction between HNRNPC1 and HNRNPG with mRNA [13, 84]. Other members of the HNRNP family, HNRNPA2/B1 could bind directly to m^6^A to regulate alternative splicing events and processing of precursor miRNAs [8].

#### Role of m^6^A in non-coding RNAs

Similar to mRNA, the presence of m^6^A on miRNA, lncRNA, and circRNA can regulate their binding to m^6^A-readers which in turn regulate their processing and maturation, abundance, translation, stability, location, function, or degradation [8–12, 99–101], but also dynamically regulate many physiological and pathological processes including tumourigenesis [102–104] (Fig. 2b). For example, the methylation of primary miRNAs (pri-miRNAs) by METTL3 increases their binding to HNRNPA2/B1, which interacts and enables microprocessor complex unit DGCR8 to target pri-miRNAs, promoting the initiation of miRNA biogenesis and their maturation into miRNAs [8, 9]. METTL14 can directly recruit DCGR8 on the m^6^A modified pri-miRNA encoding for miR-126a and consequently affecting the levels of miR-126a [12]. In addition, METTL14 has been found to be associated with chromatin during transcriptional elongation [105], which may suggest that METTL14 may contribute to the co-transcriptional recruitment of the microprocessor complex on pri-miRNA transcripts. Therefore, alternations of m^6^A levels by methylases or erasers differential expression could result in significant changes in the mature pool of miRNAs.

As recently reported m^6^A deposition can alter lncRNA stability. GAS5-AS1 transcript improves its stability by m^6^A when modulated by ALKBH5, however m^6^A can promote GAS5 lncRNA degradation through YTHDF2 and YTHDF3 binding [99, 100]. Although it is unclear whether lncRNA localization is also regulated by m^6^A, it has been shown that overexpression of METTL3 can significantly increase the nuclear localization of RP11 lncRNA [101]. The modification of m^6^A in lncRNA could as well affect their structure and influence the interactions between RNAs and between RNAs and the proteins that regulate their specific biological functions [11, 13, 14]. For instance, HNRNPC was found to bind to the m^6^A-modified hairpin compared to the unmethylated hairpin of the lncRNA MALAT1 in an “m^6^A switch”-regulated manner, which indicated that m^6^A modification disrupts lncRNA hairpin-stem structure and thus promoting its binding to HNRNPC [13]. LncRNA X-inactive-specific transcript (XIST) is methylated by METTL3, and METTL3 knockdown was shown to impair XIST-mediated transcriptional silencing of genes on the X chromosome both in vitro and in vivo [11]. Cytoplasmic lncRNA linc1281 is methylated at its 3′-end region, and the methylation marks are required for the binding of let-7 through the interaction of yet unknown proteins [14].

Regarding METTL16-mediated deposition in snRNAs, initial studies have shown to methylate A43 position of U6 snRNA, which is found near the region that base pairs with 5′ splice sites of pre-mRNAs, suggesting that METTL16 plays an important role in mRNA splicing [15].

Interestingly, ncRNAs also play significant roles in regulating the methylation levels of adenosine-6 [106, 107]. For example, in differentiating stem cells, miRNAs would regulate the binding of METTL3 to mRNAs, by using a sequence pairing mechanism, and thus modulating the abundance of m^6^A [107]. Also in hepatocellular carcinoma (HCC), the inhibition of miR-145 causes an increase in YTHDF2 expression which in turn leads to a fall in the levels of m^6^A, probably due to increased mRNA degradation [64].

While the functions of m^6^A deposition are currently not fully understood, a picture begins to emerge and shows that m^6^A methylation takes part in the regulation of mRNA processing, decay, stability, splicing, polyadenylation, nuclear export and translation. In the case of ncRNAs, m^6^A has been shown to promote their processing or enhance their functions [8, 11].

### The role of m^6^A in cancer

Numerous studies have shown that m^6^A deposition in RNA plays a critical role in many physiological processes, including circadian rhythms regulation, spermatogenesis, embryogenesis, DNA damage and stress response, pluripotency and cell reprogramming [7, 36, 41, 75, 108–110]. Furthermore, emerging evidence suggests that m^6^A regulators are also closely associated with oncogenic or tumour suppressive functions including proliferation [111], tumourigenesis [112], invasion [113], metastasis [12, 114, 115] or immune system evasion [116] in malignant tumours. Below, we summarize the main emerging roles of m^6^A writers, erasers and readers in cancer (Table 1).

**Table 1:** Alterations in N6-methyladenosine writers, erasers and readers in cancer. AML: Acute myeloid leukaemia; HCC: Hepatocellular Carcinoma; GSC: Glioma Stem Cells; LncRNA: long non-coding RNA

Factor/Enzyme	Cancer type	Alteration	Mechanism	Ref
**Writers**				
METTL3	AML	Upregulated	METTL3 promotes translation of oncogenes’ mRNAs, inhibiting cell differentiation and apoptosis.	[117, 118]
	Bladder Cancer	Upregulated	METTL3 accelerates miR221/222 maturation.	[104, 157, 321, 322]
	Breast Cancer	Upregulated	METTL3 inhibits miRNA let-7 and induces proliferation and survival.	[119]
	Colon Cancer	Upregulated	METTL3 increases miRNA-1246 expression inducing metastasis.	[120]
	Endometrial Cancer	Downregulated	METTL3 regulates the expression of members of AKT pathway and inhibits cell proliferation.	[111]
	Glioblastoma	Downregulated	METTL3 decreases oncogene expression and inhibits the self-renewal.	[35, 136]
	Glioblastoma/ Gastric Cancer	Upregulated	METTL3 increases the stability of oncogenic mRNAs.	[123, 323]
	HCC	Upregulated	METTL3 inhibits mRNA expression of tumour suppressors, increasing proliferation and metastasis.	[56, 114]
	Lung/Colon Cancer	Upregulated	METTL3 promotes oncogenes’ mRNA translation.	[113]
	Lung Cancer	Upregulated	METTL3 increases miR-143-3p expression and induces oncogenes translation.	[43, 324, 325]
	Melanoma	Upregulated	Unknown.	[326, 327]
	Osteosarcoma	Upregulated	METTL3 increases mRNA expression of oncogenes.	[328]
	Ovarian Cancer	Upregulated	METTL3 stimulates the translation of oncogenes.	[329, 330]
	Prostate Cancer	Upregulated	METTL3 increases oncogene mRNA expression.	[121]
	Prostate Cancer	Upregulated	METTL3 increases the mRNA stability of cell adhesion genes.	[122]
	Renal Cell Carcinoma	Upregulated	Unknown.	[124, 331, 332]
METTL14	AML	Upregulated	METTL14 increases oncogenes’ mRNA stability and translation.	[40]
	Bladder Cancer	Downregulated	METTL14 increases oncogenes’ mRNA decay.	[333]
	Colon Cancer	Downregulated	METTL14 regulating primary miRNA processing of YAP an SP1 pathways or downregulating lncRNA XIST.	[334, 335]
	Endometrial Cancer	Downregulated	METTL14 regulates the expression of members of AKT pathway and inhibits cell proliferation.	[111]
	HCC	Downregulated	METTL14 induces an increase of pri-miR-126 expression that suppresses tumour metastases.	[12]
	Renal Cell Carcinoma	Downregulated	METTL14 represses the translation of *P2RX6* increasing invasion.	[336]
	Pancreatic Cancer	Downregulated	Unknown.	[337]
**Erasers**				
FTO	AML	Upregulated	FTO enhances oncogenes’ mRNA stability.	[36, 37]
	Bladder Cancer	Downregulated	Unknown.	[338]
	Breast Cancer	Upregulated	FTO downregulates the expression of tumour suppressor genes.	[134]
	Cervical Cancer	Upregulated	FTO promotes translation of oncogenes, promoting migration and drug resistance.	[131, 132]
	Glioblastoma	No change	FTO regulates of oncogene expression.	[35, 136]
	HCC	Downregulated	Unknown.	[339]
	Lung Cancer	Upregulated	FTO regulates *MZF1* expression.	[133]
	Melanoma	Upregulated	FTO increases stability of critical immunotherapy resistance and pro-tumorigenic melanoma cell-intrinsic genes.	[38, 130]
	Pancreatic Cancer	Upregulated	FTO promotes oncogene mRNA stability.	[340]
ALKBH5	AML	Upregulated	Unknown.	[341, 342]
	Breast Cancer	Upregulated	ALKBH5 enhances mRNA stability of stem cell self-renewal genes.	[135, 137]
	Glioblastoma	Upregulated	ALKBH5 sustains oncogene expression.	[136]
	Gastric Cancer	No change	Binding to NEAT1 lncRNA, which promotes invasion and metastasis.	[138]
	Lung Cancer	Downregulated	ALKBH5 reduces oncogene expression and inhibits oncogenic miRNA.	[343]
	Osteosarcoma	Upregulated	ALKBH5 decreases the decay of the lncRNA PVT1.	[344]
	Ovarian Cancer	Upregulated	ALKBH5 enhances stability of oncogenes, self-renewal genes and survival genes.	[139, 142]
	Pancreatic Cancer	Upregulated	Unknown.	[140, 141]
**Readers**				
YTHDC2	Colon Cancer	Upregulated	YTHDC2 upregulates expression of pro-metastatic genes.	[143]
YTHDF1	Colon Cancer	Upregulated	Induces Wnt-β-catenin pathway and unknown.	[144, 345]
	Melanoma	Downregulated	YTHDF1 promotes the translation of tumour suppressor genes.	[146]
	Ovarian Cancer	Upregulated	YTHDF1 increases translation of oncogenes.	[145]
YTHDF2	AML	Upregulated	YTHDF2 reduces the stability of transcripts such as *TNFRSF2*.	[147]
	HCC	Upregulated	YTHDF2 inhibits *SOCS2* mRNA expression.	[56]
	HCC	Downregulated	YTHDF2 promotes the degradation of *EGFR* mRNA.	[149]
	Lung Cancer	Upregulated	YTHDF2 promotes the translation of *6PGD* mRNA.	[148]

#### Role of m^6^A writers in cancer

METTL3 and METTL14 expression have been reported to promote tumourigenesis in several cancer types (Fig. 2d). For example, METTL3 is overexpressed in acute myeloid leukaemia (AML) [117]. In this study, METTL3 was shown to methylate *BCL-2*, *PTEN*, and *c-Myc* mRNAs which resulted in their increased translation, inhibiting cell differentiation and apoptosis, and promotion leukaemia progression [117]. However, the mechanism of the m^6^A-dependent translation remains undetermined. Furthermore, another study identified that METTL3 expression in vivo was essential to maintain AML cells in an undifferentiated state, and thus maintaining myeloid leukaemia growth [118]. In comparison, METTL14 was shown to act as an oncogene in AML by increasing *MYB* and *c-Myc* mRNA stability and translation [40]. METTL3 acts as an oncogene that facilitates the growth, survival, and invasion of cells in lung and colon cancer by promoting the translation of EGFR and TAZ [113]. METTL3 is also upregulated in breast cancer, where it increases methylation and stability of *HBXIP* mRNA, which induces cell proliferation and survival of tumour cells via inhibiting the tumour suppressor let-7 g [119]. Recent studies have shown that aberrant overexpression of m^6^A modifiers is observed in colorectal cancer too. The aberrant deposition of m^6^A increased miRNA-1246 expression, resulting in the downregulation of the tumour suppressor of SPRED2 and metastasis induction [120]. Other recent studies have reported that METTL3 is overexpressed in prostate cancer cells, contributing to the growth and invasion of cancer cells through SHH-GLI1 signalling [121]. In addition, METTL3 also regulates the expression of ITGB1, thus affecting its binding to Collagen I, the mobility of tumour cells, and promoting prostate cancer bone metastasis [122].

Despite the unanimously oncogenic functions of METTL3 and METTL14 in all these cancer types, in glioblastoma and HCC several reports have demonstrated both, oncogenic and tumour suppressive roles. Initial studies showed that m^6^A methylation inhibited the growth, self-renewal, and tumourigenesis of glioblastoma stem cells by decreasing the stability and expression of key oncogenic transcripts such as *ADAM19* [35]. In contrast, in a subsequent study, METTL3 was found to be upregulated, and to be a predictor of poor patient survival. In this study, METTL3 was found to increase the stability and expression of *SOX2* mRNA, promoted the growth of glioblastoma stem cells (GSCs), and prolonged survival in mice [123]. Similarly, in HCC the expression of m^6^A writes can inversely promote or suppress tumourigenesis. For instance, METTL14 was initially identified as a tumour suppressor in HCC [12]. In this study, the authors showed that METTL14 expression induced an increase in pri-miR-126 expression, suppressing tumour metastases [12]. Conversely, in another study METTL3 was found upregulated and associated with poor prognosis in patients with HCC. In another study, METTL3 inhibited *SOCS2* mRNA expression through a m^6^A-YTHDF2 dependent manner [56]. From the reported data one can conclude that the variable expression of m^6^A writers and their bound factors, erasers and readers may account for differences in m^6^A deposition levels, as well as differences in targeted RNAs, which in turn will lead to the observed discrepancy and controversy. Yet more studies will be necessary to precisely determine the nature of METTL3 and METTL14 in glioblastoma and HCC and to identify the factors, pathways or tumour cell states responsible for the controversial findings.

In other tumours, METTL3 and METTL14 play an tumour suppressive role (Fig. 2e). For example, in endometrial cancer, 70% of tumours exhibit a reduction in m^6^A levels, either through METTL14 mutations or downregulation of METTL3 expression. METTL3-METTL14 complex loss leads to increased cell proliferation by upregulating the expression of members of the AKT pathway, and thus, showing a tumour suppressor function in endometrial cancer [111]. Similarly, in renal cell carcinoma, depletion of METTL3 promotes cell proliferation, growth, and colony formation through the PI3K-AKT-mTOR pathway activation and enhances cell migration and invasion through the epithelial-mesenchymal transition (EMT) pathway [124].

The implication of METTL16 in cancer is poorly understood, in fact only few studies have linked METTL16 to cancer [15, 125]. In recent studies, loss-of-function mutations and expression alterations were found in colon cancer, suggesting a role for METTL16 in the tumourigenesis of colorectal cancer [126, 127]. Other studies have associated METTL16 with the maturation of *MALAT1* mRNA which can act as an oncogene and a tumour suppressor in different types of cancer [125]. In addition, given the role of METTL16 in regulating snRNA methylation and hence their processing, METTL16 dysregulation could favour tumour development by inducing changes in alternative splicing. Studies in the last decade have demonstrated the potential role of alternative splicing in the aetiology of cancer [128]. Indeed, the change in the expression of key enzyme isoforms in apoptosis, metabolism, cell signalling and resistance to therapy has been attributed to the acquisition of the tumour phenotype too [128].

#### Role of m^6^A erasers in cancer

Dysregulation of m^6^A erasers have been found too associated to cancer risk, in fact FTO polymorphisms have been known to be associated to several human disorders including increased risk of cancer for decades [129]. After the discovery of FTO catalytic activity, we begin to understand the molecular mechanisms underlying FTO oncogenic activity. Initial studies showed that FTO is highly expressed in AML and exerts its oncogenic function by reducing m^6^A levels in the mRNA of the tumour suppressors *RARA* and *ASB2*, leading to inhibition of their expression [36]. Similarly, FTO was shown to induce GSCs growth, self-renewal, tumour progression, and was associated with poor survival through regulation of *ADAM19* (Fig. 2f) [35]. More recent studies have linked increased expression of FTO to other tumours. For example in melanoma, FTO expression promotes tumourigenesis and resistance to immunotherapy (anti-PD-1) by directly removing m^6^A from *PDCD1*, *CXCR4*, and *SOX10* mRNAs, thereby increasing their stability (Fig. 2f) [38, 130]. FTO is also upregulated in cervical cancer where it induces resistance to chemo-radiotherapy and enhances the response to DNA damage [131, 132]. In cervical cancer, FTO can activate the β-catenin pathway and increase ERCC1 expression that is associated with worse prognosis. Furthermore, FTO promotes cell migration and proliferation by positive regulation of *E2F1* and *MYC* [131, 132]. In lung cancer FTO is found overexpressed and is associated with poorer prognosis, facilitating cell proliferation and invasion, and inhibiting apoptosis by regulating MZF1 expression (Fig. 2f) [133]. In breast cancer too, high levels of FTO promotes cell proliferation, colony formation and metastasis in vitro and in vivo through downregulation of *BNIP3* expression [134]. The compelling evidence clearly indicates a role for FTO in cancer, yet although FTO has been described to remove m^6^A in the mRNA of tumour suppressors or genes that could confer resistance to immunotherapy, currently there is insufficient evidence to confirm that the effects detected in cancer are due exclusively to its demethylase activity.

Aberrant overexpression of ALKBH5, the second m^6^A demethylase to be identified, has been also associated to several cancer types (Fig. 2f) [135–142]. ALKBH5 has been found overexpressed in glioblastoma and its expression is associated with poorer prognosis, and it promotes GSCs proliferation and tumour progression by enhancing *FOXM1* expression [136]. In breast cancer, ALKBH5 has been shown to promote tumourigenesis by decreasing adenosine methylation in *KLF4* and *NANOG* mRNAs, enhancing their stability in breast cancer stem cells [135, 137]. In gastric cancer, it promotes invasion and metastasis by demethylating the lncRNA *NEAT1* [138]. ALKBH5 expression is found increased in ovarian cancer too and its expression correlates with poorer survival. The upregulated expression promotes proliferation, invasion and autophagy through the mTOR and BLC2-Beclin1 pathways [139, 142]. Similar to other m^6^A regulators, ALKBH5 has been demonstrated to have a tumour suppressive role in other tumours (Fig. 2g). For example, in pancreatic cancer cells, loss of ALKBH5 induces increased methylation of the lncRNA *KCNK15-AS1*, leading to its downregulation and increased cell migration. ALKBH5 in pancreatic cancer also has been shown to inhibit tumourigenesis by reducing WAF-1 levels and hindering Wnt signalling activation [140, 141].

#### Role of m^6^A readers in cancer

m^6^A readers are also aberrantly expressed in cancer, and their defective function has been linked to oncogenic or tumour suppressive roles, however we begin to appreciate only now the interplay of these RNA binding proteins, m^6^A and cancer. YTHDC2 and YTHDF1 are overexpressed in colorectal cancer, both are associated with poor prognosis in patients and high cell proliferation and metastatic potential. In these tumours, YTHDC2 regulates the expression of tumour promoter genes such as *HIF1A* [143]. Silencing of YTHDF1 inhibits tumourigenicity in vitro and tumour growth in vivo by inhibiting the Wnt-β-catenin pathway and its expression induces resistance to chemotherapy [144]. YTHDF1 is also upregulated in cancer. In particular, in ovarian cancer YTHDF1 increases *EIF3C* translation, facilitating tumourigenesis and metastasis [145]. In contrast, YTHDF1 acts as a tumour suppressor in melanoma where it promotes the translation of the tumour suppressor *HINT2*, thus inhibiting tumour development [146]. In the case of YTHDF2, its overexpression in AML has been shown to reduce the half-life of various m^6^A-containing transcripts which are involved in TNF signalling and whose upregulation promotes cell apoptosis [147]. Furthermore, YTHDF2 was found to be upregulated in lung cancer, and to aberrantly promote the translation of *6PGD* mRNA which is critical for the promotion of tumour growth [148]. Nevertheless, YTHDF2 is also capable to suppress cell proliferation, tumour growth and activation of MEK and ERK signalling via promoting the degradation of *EGFR* mRNA in HCC cells [149].

### Targeting m^6^A machinery in cancer

Considering the critical roles of the m^6^A regulatory proteins in several cancer hallmarks, they are promising therapeutic targets, specially the writers and erasers, since their activity can be modulated by small molecules. Although there are currently no small molecule inhibitors of RNA methyltransferases, several demethylase inhibitors have been discovered or developed by biochemical- or cell-based small-molecule compound library screening or chemical synthesis. Most commonly, those developed or discovered inhibitors target FTO. In a seminal study, *Su R* et al. FTO was found to be inhibited by the oncometabolite R-2-hydroxyglutarate (R-2HG) [37]. In this study, R-2HG was used to directly inhibiting FTO in AML and glioma cells, which resulted in increased methylation and decreased expression of *c-MYC* and *CEBPA* mRNAs, blocking proliferation, cell cycle, and inducing apoptosis in AML and glioma cells [37]. Since then, other studies have attempt to develop small-molecule inhibitors to target FTO or AKLBH5 RNA demethylases, given promising results at the pre-clinical level. For example, the ethyl ester form of Meclofenamic acid (MA) MA2, a US Food and Drug Administration (FDA)-approved nonsteroidal anti-inflammatory drug, was found to be a FTO inhibitor which led to elevated levels of m^6^A modification in mRNAs in glioblastoma cells, suppressing tumour progression and prolonging the lifespan of GSC-grafted mice [35, 150]. In addition, based on the structures of FTO and ALKBH5 domains other groups have designed 2-oxoglutarate and iron-dependent oxygenases (2OGX) inhibitors to target m^6^A erasers [151], such as for example the IOX3 inhibitor [152]. However the promising inhibitors have some limitations and need to be considred before their use in the clinic. All 2-oxoglutarate derivates are not selective and may also suppress the activity of other Fe (II) and 2OG dependent oxygenases. More recent studies have developed two FTO inhibitors, namely FB23 and FB23–2, which have been shown to suppress proliferation and promote AML cell differentiation/apoptosis in vitro and significantly inhibit the progression of human AML in xenotransplanted mice [153].

Despite the contradictory results for some types of tumours, m^6^A regulators have shown to have wide implications in several cancer hallmarks. The controversial results reflect however that the consequences of aberrant m^6^A deposition may dependent on the cancer or cell type context, dysregulation of other signalling pathways and distinct set of substrates. Thus, future studies will accurately determine the biological function of each individual m^6^A regulator in different types of cancer, and they will identify each critical target transcript revealing the exact underlying mechanism. In addition, development of selective and clinically effective inhibitors for m^6^A regulatory enzymes may provide effective therapeutic strategies, alone or in combination with other therapeutic agents. For example, neoantigen-specific immunity was shown to be regulated through YTHDF1 in a m^6^A-dependet manner. Mechanistically, transcripts encoding lysosomal proteases were shown to be marked by m^6^A and bound by YTHDF1, leading to increased translation of lysosomal cathepsins and decreased cross-presentation of dendritic cells, implicating YTHDF1 and m^6^A as potential therapeutic targets in anticancer immunotherapy [116].

## 5-methylcytosine

### m^5^C deposition in RNA

m^5^C is a conserved and prevalent mark in RNA in all life domains. m^5^C is found in a wide range of RNAs but it is most abundant in eukaryotic tRNAs and rRNAs (extensively reviewed in [16]). Current global m^5^C detection methods rely on the chemical reactivity of cytosines to be deaminated in the presence of sodium bisulphite, or immunoprecipitation methods that use either antibodies against m^5^C or RNA methyltransferases previously crosslinked to the RNA target (extensively reviewed in [16]). RNA bisulphite sequencing is the most common used technique to map m^5^C and while it has resulted effective in detecting m^5^C in abundant RNAs such as tRNA and rRNA [24, 42]. In mRNA different studies obtained very different results. From studies identifying m^5^C sites in 8000 RNAs [154], to other studies finding only a few methylated mRNAs [155]. These controversial findings have raised the need to develop more robust methods for truly identifying m^5^C depositions in mRNA [156]. More recent studies have reported only few hundred m^5^C sites in human and mouse transcriptomes using an improved bisulphite sequencing method and a novel computational approach (Fig. 1) [156, 157]. What is still undetermined is whether this low prevalence has biological value and it needs to be further investigated.

#### m^5^C writers

**Fig. 3:**
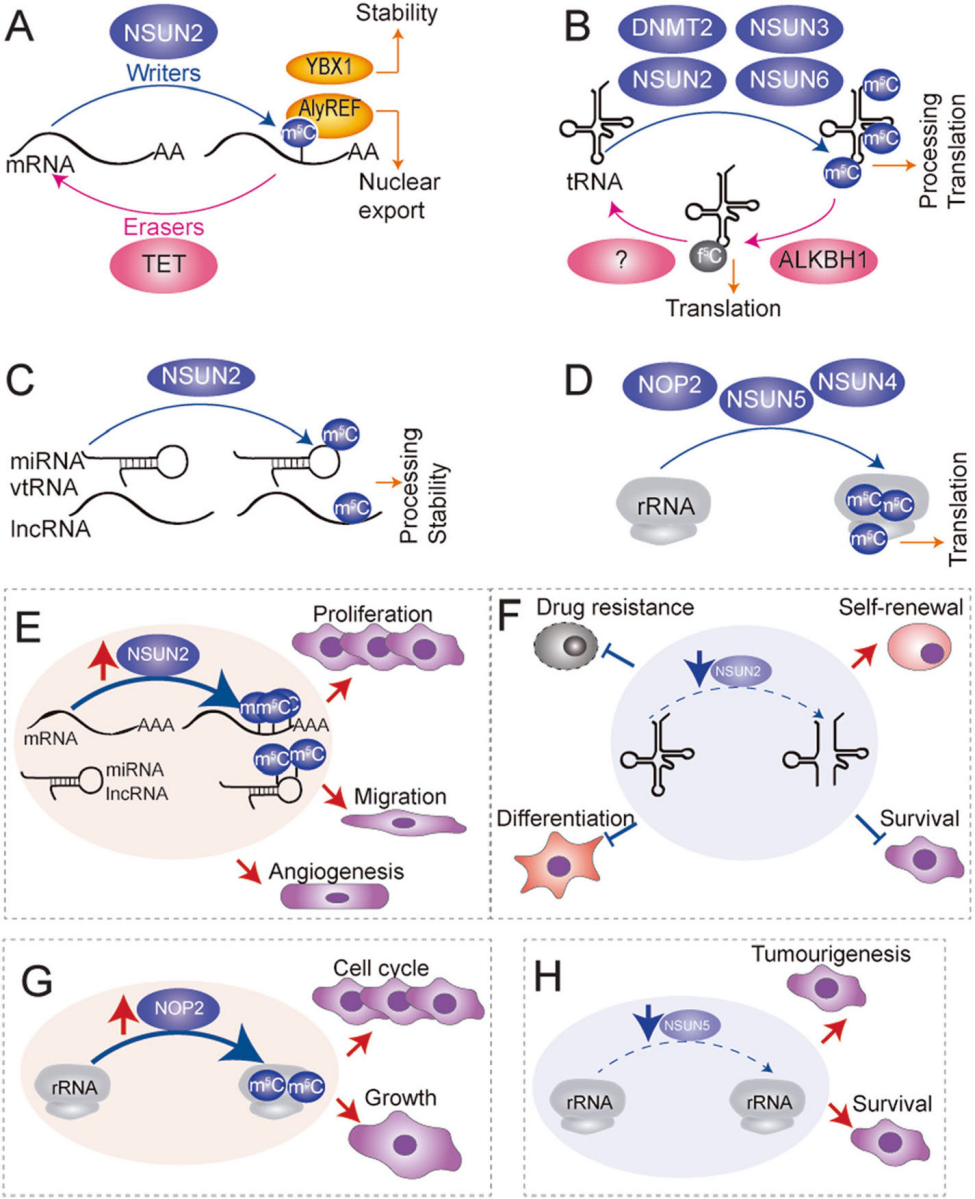
Molecular mechanism of m^5^C deposition in RNA, biological function implication in human cancer. **a**-**d** m^5^C RNA deposition landscape in mRNA (**a**), tRNA (**b**), rRNA (**d**), and ncRNAs (**c**) mediated by writers (blue balloons) NSUN2–6, DNMT2 and NOP2, erasers (pink balloons) TET and ALKBH1, and reader proteins (yellow balloons) YBX1 and AlyREF, and their role in mRNA metabolism. **e**-**h** Aberrant m^5^C deposition in RNAs promotes or suppresses tumour progression through NSUN2 upregulation in mRNA, miRNA and lncRNA (**e**), NSUN2 downregulation in tRNA (**f**), NOP upregulation in rRNA (**g**) or NSUN5 downregulation in rRNA (**h**). Red arrows indicate induction. Blue arrows with flat end represent inhibition

**Fig. 4:**
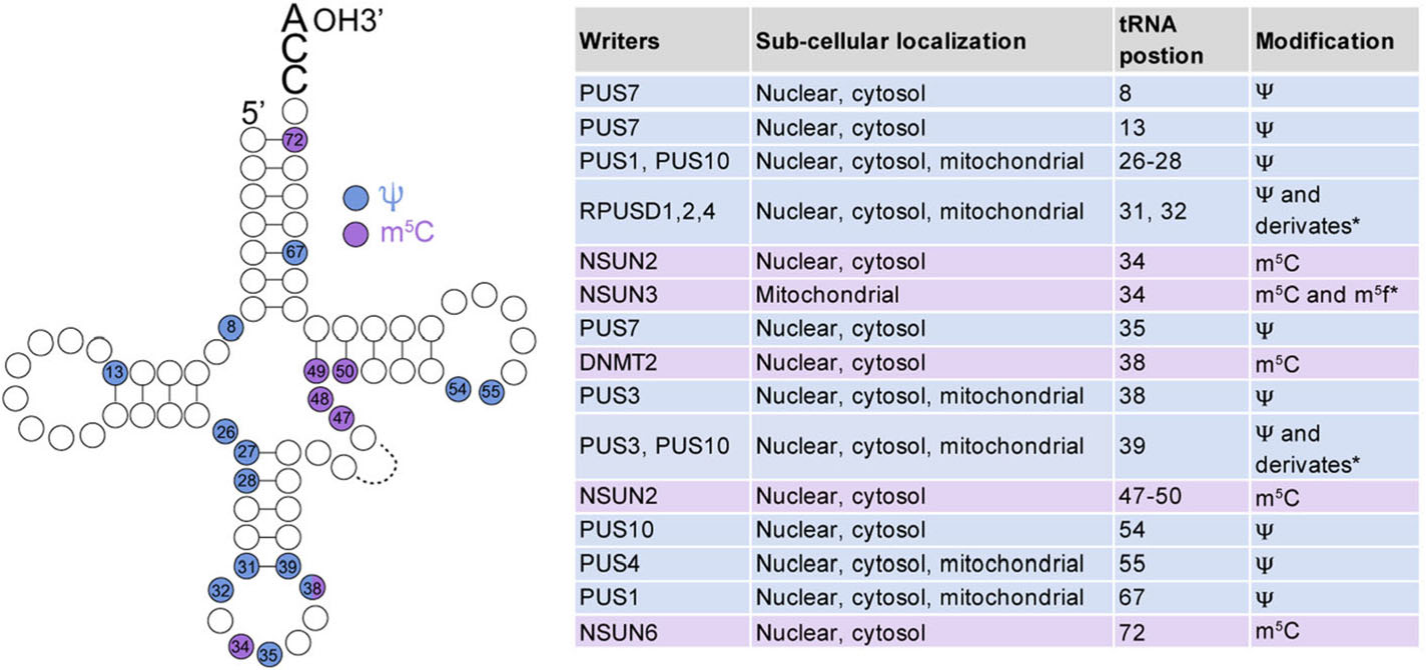
Simplified structure of a tRNA and all known Ψ and m^5^C events in humans. Ψ modification is shaded in blue and m^5^C in purple. The table on the right indicates the position and modification in tRNA, the human enzyme that has been identified to catalyse this modification, the sub-cellular localization of tRNAs containing the modification. m^5^C, 5-methylcytosine; f^5^C, 5-formylcytosine Ψ, pseudouridine; Ψ derivates: Ψm, 2ʹ-O-methylpseudouridine and m^1^Ψ, 1-methylpseudouridine. Proteins: PUS, pseudouridine synthase; RPUSD, RNA pseudouridine synthase domain-containing protein; NSUN, NOL1/NOP2/SUN domain family member; DNMT2, DNA methyltransferase 2. f^5^C* is formed from m^5^C by ALKBH1 (Alpha-Ketoglutarate-Dependent Dioxygenase AlkB Homolog 1) in mitochondrial tRNAs. Ψ derivates are formed from Ψ by methyltransferases

In humans, cytosine-5 methylation is catalysed by the NOL1/NOP2/sun (Nsun) family and the DNA methyltransferase member 2 (DNMT2, TRNA Aspartic Acid Methyltransferase 1 or TRDMT1) [158, 159]. DNMT2, NSUN2, NSUN3 and NSUN6 all methylate cytoplasmic tRNAs, yet with different specificity and at different residues (Figs. 3b, 4) [42, 160–167]. For example, NSUN2 methylates the vast majority of tRNAs at the variable loop, and in leucine at the wobble position [42, 168, 169], DNMT2 methylates three tRNAs at the anti-codon loop [161, 162, 170], and NSUN6 targets the acceptor stem of few tRNAs [164]. NSUN2 methylates also mRNA, ncRNAs and lncRNAS (Fig. 3a, c) [157, 168, 171–173]. NSUN3 targets tRNAs in the mitochondria, and its deposition is required for the formation of 5-formylcytosine (f^5^m) [160, 163, 166] (Figs. 3b, 4). NOP2 (NSUN1) and NSUN5 are nucleolar and methylate very conserved residues in 28S rRNA, close to the peptidyl-transferase centre [165, 174, 175] and at the interface between the large and small subunit respectively (Fig. 3d) [165, 174, 175]. Finally, NSUN4 targets the small subunit of mitochondrial rRNA (Fig. 3d) [176].

#### m^5^C erasers

While writers for m^5^C are now well documented, the existence of m^5^C erasers are still under debate. Some reports have however indicated that m^5^C can be oxidised to generate 5-hydroxymethylcytosine (hm^5^C) by enzymes of the ten-eleven translocator family (TET) in mRNA (Fig. 3a) [177, 178] and the formation of f^5^C by Alpha-Ketoglutarate-Dependent Dioxygenase AlkB Homolog 1 (ALKBH1) at the wobble position of mitochondrial tRNAs (Fig. 3b) [160, 163, 166]. While the biological relevance of f^5^C deposition in mitochondrial tRNAs has been well established [160, 163, 166], the biological relevance of the low abundant hm^5^C deposition in mRNAs remains yet to be determined.

### Molecular function of m^5^C deposition in RNA

#### Role of m^5^C in non-coding RNAs

The functional consequences of m^5^C loss in tRNAs have been well documented. Deposition of m^5^C by DNMT2 and NSUN2 protects tRNAs from endonucleolytic cleavage by angiogenin [42, 162, 167]. NSUN2-mediated m^5^C deposition in tRNAs regulates differentiation and stress responses in tissue and in cancer stem cells [33, 42, 179–181]. Molecularly, 5-cytosine methylation of tRNA protects them from processing into tRNA-derived small RNA fragments (tRFs) by angiogenin [42, 162, 167, 170, 182]. The formation of tRFs is usually induced under stress and can repress canonical translation and favour ribosome assembly in unconventional 5’ start sites found in 5’ UTR of stress response transcripts [183]. In contrast, loss of DNMT2-mediated methylation of tRNA at C38 causes tRNA cleavage which leads to specific codon mistranslation [167]. Similarly, deletion of rRNA cytosine-5 methyltransferases in yeast, flies, worms, and mice are not lethal, however, in all cases, the level of m^5^C deposition plays a significant role in regulating the cellular response to stress including drugs, DNA damage, oxidative stress, or environmental cues [23, 175, 184]. Mechanistically, rRNA modifications such as NSUN5-mediated methylation fine-tune the translation capacity of the ribosome by adapting it to specifically translate mRNAs relevant in stress [24]. m^5^C has also been detected in small ncRNAs and lncRNA [154, 168, 169, 185–187]. m^5^C deposition on ncRNA such as vault RNA also affects its processing into miRNA-like regulatory small RNA [168, 171].

#### Role of m^5^C in mRNAs

Only two recent studies have reported finding readers that bind to m^5^C-modified mRNAs, revealing the biological relevance and the role of m^5^C deposition in mRNAs (Fig. 3a). In one report, nuclear mature methylated mRNAs at any nucleotide position interacted with the nuclear export factor AlyREF and were more likely to be exported to the cytoplasm [173]. More recently, another report showed that in cancer cells NSUN2 aberrant methylation of oncogenic mRNAs at the 3′ UTR increased their interaction with the reader protein Y-box-binding protein 1 (YBX1), which maintained the stability of its targeted mRNAs by recruiting ELAVL1 [157]. Despite these studies, the advances in finding the prevalence, deposition site preference, molecular function, writers and readers of m^5^C on mRNA still remain elusive.

### The role of m^5^C in cancer

#### Role of m^5^C writers in cancer

Over the past decade, a number of cytosine-5 methyltransferases have been found to be associated with various human diseases including several cancer types (Table 2). NOP2 overexpression was long shown to increase proliferation of mouse fibroblasts (Fig. 3g) [188], and it was found to be a valuable proliferation marker [189]. NOP2 expression has been found to be upregulated in breast, lung, prostate cancer, and gallbladder carcinoma, and its expression correlates with poor prognosis [190–192]. Mechanistically, NOP2 has been shown to bind to the T-cell factor (TCF)-binding element of the cyclin D1 promoter, recruiting TERC elements (telomerase RNA component) and activating cyclin D1 transcription. Whether NOP2’s methylating activity of rRNA is involved remains unexplored [193]. NSUN2 expression alterations have been linked to several cancer types including breast, skin, colon, ovarian, oesophageal, bladder, gallbladder, gastric cancer, and head and neck squamous carcinoma [157, 172, 194–200]. DNMT2 is upregulated in hundreds of tumour samples and several somatic mutations in *DNMT2* have been identified in different tumours types [201]. NSUN4 loci is associated to increase risk of breast, ovarian or prostate cancer and its high expression is associated with HCC [202, 203]. Lastly, NSUN5 expression has been found to correlate with poor survival in glioblastoma [24] and NSUN7 high expression is associated to shorter survival in low-grade gliomas [204].

**Table 2:** Role of aberrant deposition of m^5^C in cancer. AML: Acute myeloid leukaemia; ALL: Acute lymphoblastic leukaemia; HCC: Hepatocellular Carcinoma

Factor/Enzyme	Cancer type	Alteration	Mechanism	Ref
**Writers**				
NOP2	Breast cancer	Upregulated	Unknown.	[190]
	Leukaemia	Increased	NSUN1 mediates chromatin structures that modulate 5-AZA resistance.	[216]
	Lung adenocarcinoma	Upregulated	Unknown.	[192]
	Prostate cancer	Upregulated	Unknown.	[191]
NSUN2	Bladder cancer	Upregulated	NSUN2 targets HDGF 3' UTR.	[157]
	Skin, breast and colon cancer	Upregulated	Unknown.	[194]
	Squamous cell carcinoma	Upregulated	Protects tRNA from cleavage and increases cell survival	[33]
	Gallbladder carcinoma	Upregulated	NSUN2 interaction with RPL6.	[197]
	Gastric cancer	Upregulated	Repressing p57(Kip2) in an m^5^C-dependent manner.	[200]
	Head and neck squamous carcinoma	Upregulated	Unknown.	[199]
	Oesophageal squamous cell carcinoma	Upregulated	Increased methylation and stability of NMR lncRNA.	[172]
	Ovarian cancer	Upregulated	Unknown.	[195]
	Several cancers	Increase copy number	Unknown.	[198]
NSUN3	Leukaemia	Undetermined	NSUN3 direct binding to hnRNPK.	[216]
	Lung Cancer	Genomic aberrations	Unknown.	[346]
NSUN4	Breast, ovarian, and prostate cancer	Susceptibility Loci	Unknown.	[202]
	HCC	High expression	Methylation processes.	[203]
NSUN5	Glioblastoma	Downregulation	Ribosome structural changes that lead to stress adaptive translational programmes.	[24]
NSUN7	Low Grade Gliomas	High expression	Unknown.	[204]
DNMT2	Leukaemia	Undetermined	DNMT2 direct binding to hnRNPK.	[216]
	Several cancer types	Upregulated and somatic mutations	Inhibition of m^5^C deposition in tRNAs.	[201]
**Erasers/5-hydroxymethylcytosine writers**				
TET1	Glioblastoma	Upregulated	Unrelated to RNA hydroxymethylation.	[213]
TET2	Glioblastoma	Downregulated	Unrelated to RNA hydroxymethylation.	[214]
	AML	Point mutations	Unknown.	[210]
TET3	Glioblastoma	Downregulated	Unknown.	[215]
TET Family	Hematologic malignancies	Several	Unrelated to RNA hydroxymethylation.	[212]
TET1/TET2	HCC	Downregulated	Unrelated to RNA hydroxymethylation.	[347]
ALKBH1	ALL	Upregulated	Unknown.	[211]
	Gastric cancer	Upregulated	Unknown.	[348]
**Readers**				
YBX1	Bladder cancer	Upregulated	YB1 translocation to the nucleus induces acquisition of drug resistance by upregulating expression of multidrug resistance-1 (MDR-1) gene.	[349]
	Breast cancer	Upregulated	YB1 interacts and inhibits ESR1-FOXA1 complex.	[350]
	Several cancer types	Upregulated	Multifunctional oncoprotein.	[351]
ALYREF	HCC	High expression	Cell cycle regulation and mitosis.	[203]
	Oral squamous cell carcinoma	High expression	Unknown.	[352]
	Several cancer types	High expression	Unknown.	[353]

Regarding the associated mechanisms, the methylases’ molecular role seems to be tissue and context dependent, showing different unique substrates preferences for each cancer type, but with a unifying oncogenic result. For example for NSUN2, in most reports its overexpression is shown to regulate the fate of one single transcript of tumour suppressor genes or oncogenes and thus promoting proliferation [205]. Very recently NSUN2 was also shown to promote tumour progression by methylation of *NMR* ncRNA in oesophageal cancer [172]. In another recent study, it was reported that aberrant NSUN2-mediated methylation at the 3′ UTR of oncogenic mRNAs such as heparin-binding growth factor (*HDGF*) mRNA can increase their stability by interacting with YBX1 (Fig. 3e) [157]. While all these reports focused on the fate of mRNAs or other transcripts whose m^5^C prevalence is low, and considering that tRNAs are the main NSUN2 targets and are highly methylated at C-5, it is not yet clear whether the potential role of NSUN2 in cancer might actually be mediated by modifications of mRNA.

Regarding tRNA methylation functions, NSUN2 has been reported to be upregulated in a population of proliferative progenitor cells in skin tumours that rely on the correct deposition of m^5^C onto tRNAs [33]. Unexpectedly, deletion of *NSun2* in mouse cells and mouse cancer cells led to hypomethylated tRNAs which showed to be more vulnerable to the ribonuclease angiogenin, and consequently to the accumulation of 5′-tRNA fragments [33, 42, 182]. Notably, the increase cleavage of tRNAs did not resulted in depletion of mature tRNAs, supporting that cleaved tRNAs mediated the phenotypic consequences. In fact, there is a growing appreciation that biogenesis of tRNA fragments is largely induced under stress conditions and their aberrant expression, commonly found in cancer, may indicate important regulatory functions in tumourigenesis (extensively reviewed in [183]). The understanding of their function is still in its infancy, but the reported data indicate that 5′-tRNA fragments can reprogram translation to favour stress responses by regulating the binding of translation initiation factors to the translation initiation complex [206–208]. Indeed, in *Nsun2* deficient mouse cancer cells, ribosome profiling data showed an increased translation of genes associated to stress response pathways and decreased translation of genes associated to differentiation [33]. Importantly, the data showed that *Nsun2* deficiency in cells blocked them in an undifferentiated and more proliferative state necessary for the self-renewal of tissue or tumour stem cells [33, 179, 181, 182]. However, prolonged deficiency showed to increase the sensitivity to stress of the undifferentiated cells [33, 42]. The finding was further supported by showing that *Nsun2* deficient skin tumour initiating cells were more efficiently killed by using chemotherapeutic agents such as 5′ FU or cisplatin, which could be further rescued upon treatment with angiogenin inhibitors [33]. Although the exact mechanism by which 5′-tRNA fragments directly favoured translation of specific set of transcripts is still unknown, the study demonstrated that the combinatory use of tRNA fragment biogenesis enhancers such as NSUN2 inhibitors, with convectional chemotherapeutic agents could result in an efficient strategy to specifically eliminate tumour initiating cells. Thus, these and other studies show that tumour initiating cells and cancer cells require tight control of tRNA methylation, tRNA fragment biogenesis and protein synthesis [209] for accurate cell responses and to maintain the bulk tumour, and suggest the use of tRNA methylation inhibitors as potent cancer initiating cells sensitizers to cytotoxic stress (Fig. 3f).

The contribution of tRNA modifications in survival and differentiation is further supported by findings that *Dnmt2* deletion in mice and flies was characterized by defects in stress responses and differentiation [162, 167]. In line with *Nsun2 −/−* mice phenotype, Dnmt2-depletion in mice did not globally perturb protein synthesis rates but rather affected specific mRNAs through reduced translation fidelity caused by loss of tRNA methylation [167]. Yet the potential biological impact of elevated tRNA cleavage due to Dnmt2 loss or cytosine-5 methylation inhibition remains completely unexplored. Additional work will be necessary to unveil the contribution of tRNA fragment abundance to the complex phenotypes observed.

Alterations in rRNA methylation have been also linked to cancer. *NSUN5* mRNA expression is strongly associated with poor survival in glioblastoma patients. Epigenetic loss of NSUN5 expression in gliomas leads to rRNA cytosine hypomethylation and to increased translation of survival factors, rendering glioma cells sensitive to substrates of the stress-related enzyme NAD(P)H quinone dehydrogenase 1 (NQO1) (Fig. 3h) [24].

Thus, these findings highlight the importance of the tight control of tRNA and rRNA methylation and specialised protein synthesis programmes and set the basis to search for tRNA methylase inhibitors as a potent new approach to treat cancer.

#### Role of m^5^C erasers in cancer

5-hydroxymethylation writers have been found altered in cancer too. *TET* and *ALKBH1* mutations or expression alterations are highly associated to some malignancies. For example, *TET*2 and *ALKBH1* mutations have been associated to lymphoblastic and myeloid leukaemia [210–212]. TET1 expression is upregulated in glioblastomas [213], TET2 is downregulated in gliomas [214], and TET3 is epigenetically repressed in gliomas [215]. While mechanistically the cause has been always associated to DNA demethylation or hydroxymethylation defects, TET and ALKBH1 have been shown to oxidise m^5^C in RNA too, raising the question as to whether defects of RNA hydroxymethylation could be also linked to cancer.

### Targeting m^5^C machinery in cancer

To date no specific cytosine-5 RNA methyltransferase inhibitor has been developed. Yet, given that several drugs designed to interfere with cytosine-5 methylation of DNA rely on the use of chemical analogues of cytosine, they may well interfere with RNA methylation [50, 216]. In fact, in a study by Lyko and co-workers, complete inhibition of Dmnt2-mediated 5-cytosine tRNA methylation with azacytidine in cancer cells reduced their proliferative capacity, supporting the notion that reduced cytosine-5 methylation of tRNAs may be an efficient cancer therapeutic strategy [50]. Despite the promising results and potential clinical implications, the fact that those analogues can both inhibit RNA and DNA methyltransferases raise awareness of the use of this unselective drugs, which may affect the methylation of multiple targets (DNA, tRNA, rRNA, mRNA, ncRNA) and may have devastating consequences.

In line with the notion that inhibition of tRNA methylases may contribute to chemotherapy resistance, silencing of other tRNA methyltransferases such as the 7-guanosine methylase METTL1 which also methylates tRNAs at the variable loop of several tRNAs has shown to increase sensitivity of cancer cells to 5’FU [217].

## Pseudouridine

### Pseudouridine deposition in RNA

Pseudouridine (Ψ), the C5-glycoside isomer of uridine, was the first posttranscriptional modification discovered and is one of the most abundant modifications of RNA [218, 219]. Despite its discovery over seventy years ago, we are only now beginning to uncover its biological function [25–27, 220–223].

Pseudouridine was initially detected on yeast tRNAs and rRNA [224, 225], and now we know that different types of RNAs including tRNA, rRNA, small nuclear RNAs (snRNAs), small Cajal Body-specific RNAs (scaRNAs), miRNAs, lncRNAs are also Ψ-modified [226]. Most importantly, with the current technological advents which rely on chemical treatment of RNA using soluble carbodiimide(N-Cyclohexyl-N′-(2-morpholinoethyl) carbodiimide metho-ρ-toluenesulfonate (or CMCT) for the generation of reverse transcription-stops, several groups have successfully performed genome-wide mapping experiments validating established targets and revealing novel substrates like mRNAs (Fig. 1) [25, 26, 221]. Since then, several seminal studies have followed discovering novel substrates and molecular roles of Ψ [25–27, 221, 222, 227–230].

#### Pseudouridine synthetases

**Fig. 5:**
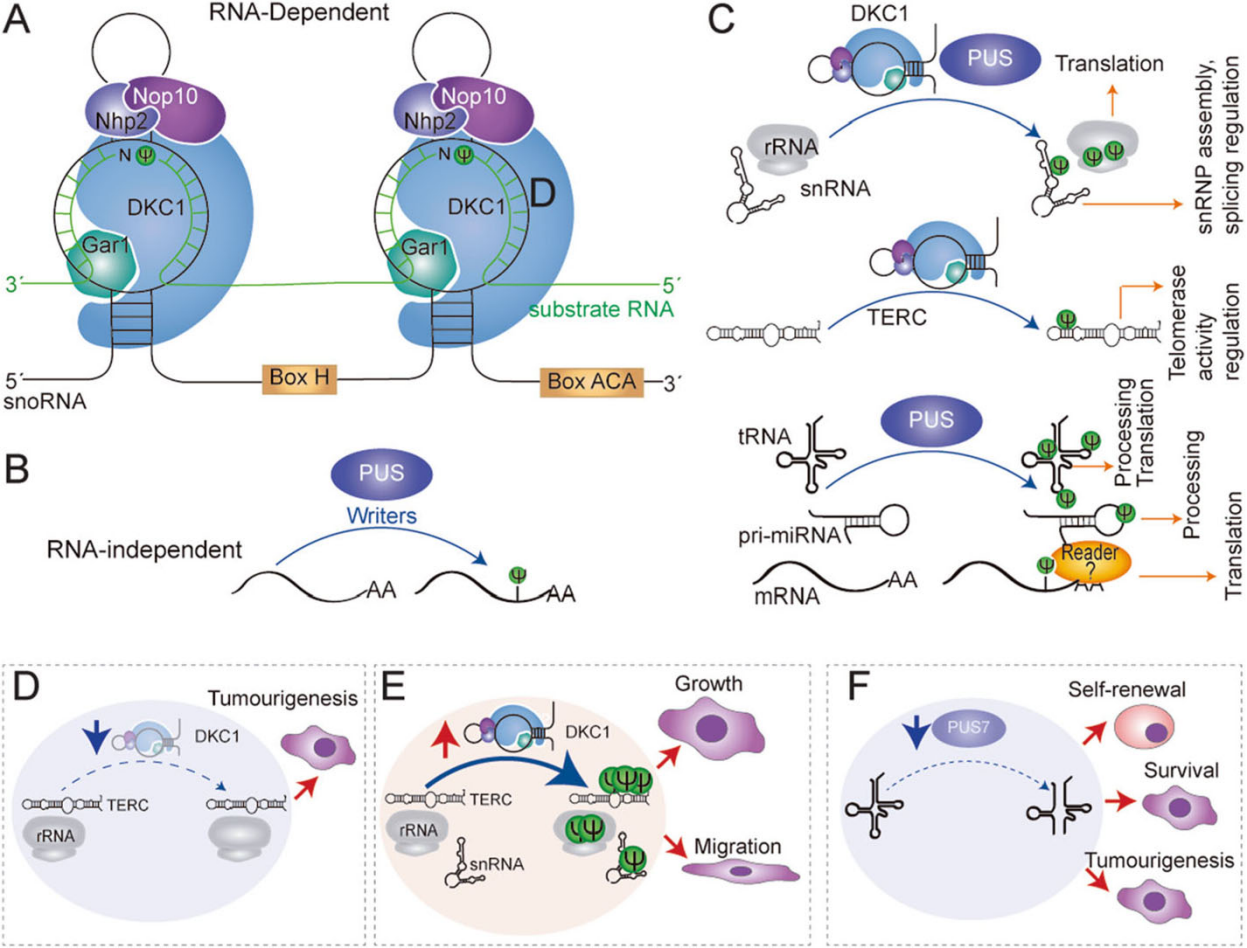
Ψ deposition mechanisms and pathological implications in cancer. **a** Schematic illustration of RNA-dependent pseudouridylation mechanisms. Substrate recognition is achieved by sequence and structure homology of the substrate with the structural stems and loops formed by box H/ACA small nucleolar RNAs (snoRNA). Dyskerin (DKC1) is the catalytic unit and non-histone protein 2 (Nhp2), nucleolar protein 10 (Nop10) and glycine-arginine-rich protein 1 (Gar1) are regulatory units. The RNA substrate is represented in green. **b** Illustration of RNA-independent pseudouridylation mechanisms. Substrate recognition and catalysis are performed by one single pseudouridine synthases (PUS) (blue). **c** Representative scheme illustrating the target specificity for each pseudouridine synthases. The fate of each modified RNA is also illustrated with orange arrows. Pseudouridylated RNAs may be also recognised by still unknown readers (?). **d** Altered expression of pseudouridine synthases can lead to cancer. For example, reduced expression of DKC1 induces a reduction of pseudouridylation in TERC and rRNA, leading to dysfunctional TERC and rRNA and increasing tumourigenesis. **e** In glioma, An increased expression of DKC1 can lead to an increased Ψ deposition on rRNA, snRNA and TERC, and thus promoting cancer cell growth and migration. **f** PUS7 decreased expression leads to hypomodified tRNA-derived fragments, leading to increased self-renewal and survival in bone marrow mononuclear cells, promoting tumourigenesis. Red arrows indicate an increase and blue arrows a decrease of processes or enzymes

Pseudouridylation can be achieved through two distinct mechanisms, namely RNA-independent and RNA-dependent pseudouridylation. The RNA-dependent mechanism relies on RNA–protein complexes known as box H/ACA small ribonucleoproteins (snoRNPs), which consist of a box H/ACA snoRNA and four core proteins; dyskerin (also known as NAP57 or DKC1), non-histone protein 2 (Nhp2), nucleolar protein 10 (Nop10) and glycine-arginine-rich protein 1 (Gar1). In H/ACA ncRNA is responsible for substrate recognition through complementary base-pairing interactions with the RNA substrate, and the catalytic activity is provided by DKC1 (Fig. 5a) (reviewed in [227]). The RNA-independent pseudouridylation is catalysed by a single enzyme, pseudouridine synthases (PUS), which carry both substrate recognition and catalysis without an RNA template strand (Fig. 5b). The rules governing RNA substrate recognition by RNA-independent Ψ synthases have only been elucidated in a few cases (for a review, see [231]). Some of the characterized Ψ synthases have a rather strict substrate specificity and are only able to modify one position in only one type of cellular RNA, such as, for instance, tRNAs. The strict substrate specificity depends on the universally conserved G^53^UUCNANNC^60^ sequence in most tRNAs, and on the three-dimensional structure of the TΨC loop [232]. Similar results were found by analysing the co-crystal structures of the prokaryotic enzyme TruB with isolated tRNA stem-loops [233]. The crystals illustrated how the enzyme core domain makes extensive interactions with the RNA, and how TruB requires the consensus sequence around the TΨC loop [233]. The mechanism for enzymes with a broader susbtrate spectrum is different. For example, PUS1, which methylates three positions in tRNAs, relies on two positively charged RNA-binding clefts along the surface of the protein which uniquely interacts with the tRNA [234]. In the case of PUS7, the sequence and two-dimensional structure are required for substrate recognition [235]. How other Ψ synthases with a broader substrate repertoire can recognize all the different substrates with a high degree of specificity is still under study.

In eukaryotes, there are over fourteen different PUS enzymes, ten of these enzymes belong to the PUS family, including PUS1-10 [227, 236]. Each of them has specific substrates, but also share some of the substrates (Fig. 5C). For example, PUS1 modifies tRNAs at positions 38, 39 and/or 40 [234]. PUS4 [237] and PUS10 modify too tRNAs, but at position 55 [238]. Regarding erasers, an important difference between pseudouridylation and base methylations is that pseudouridylation is an irreversible modification in mammals, and instead mammals excrete the intact nucleoside [239]. No readers have been described to date.

### Molecular function of Ψ deposition in RNA

Pseudouridylation plays different physiological roles depending on the RNA that is modified, but most commonly experimental data confirmes an important role in different aspects of gene expression regulation. The presence of Ψ is capable of increasing the rigidity of the phosphodiester backbone of the RNA and affecting its thermodynamic stability and spatial conformation, making short RNAs more stable [240, 241]. In snRNAs, in vitro pseudouridylation generates RNA conformational changes that influences the snRNA-mRNA interactions [242, 243]. In vivo studies have shown that these conformational changes are important for the snRNA activity. For example, the yeast U2 snRNA is pseudouridylated during stress at positions U56 and U93 by the RNA-independent enzyme PUS7 and a box H/ACA RNP complex (snR81), having functional implications in the efficiency of pre-mRNA splicing [229]. Ψ’s role in small nuclear RNAs was thoroughly reviewed recently in [220].

Pseudouridylation also generates structural stability to different types of RNA, such as rRNAs, which is necessary for their function [244, 245]. In rRNAs, Ψs are found at the decoding site, mRNA channel, peptidyl transferase centre, tRNA binding site and ribosomal subunit interface, thus showing an important role for the correct assembly and function of the ribosome and for protein synthesis [246]. For example in yeast, alterations or substitutions of amino acids in the Ψ synthase domain of Cbf5p abolish pseudouridylation of rRNA, resulting in growth defects and reduced levels of cytoplasmic 40S and 60S subunits of rRNA [244]. In mouse embryonic fibroblast cells, expression of dyskerin mutants leads to altered rRNA processing, unstable secondary structure of rRNA and cell growth defects [245]. Another similar example occurs upon the loss or disruption of H/ACA snoRNAs that guide rRNA modification. Pseudouridylation at position U2919 in yeast rRNA is guided by five H/ACA snoRNAs, whose loss leads to reduced rRNA pseudouridylation at U2919, and impaired 18S rRNA biogenesis and growth defects in yeast [247].

In mRNAs, the incorporation of Ψ can mediate nonsense-to-sense codon conversion facilitating the base pairing in the ribosome decoding centre, and thus resulting in protein diversity [248]. Other in vitro studies have also reported that translation of mRNAs containing pseudouridines is slowed down and mRNA decoding affected compared to non-modified mRNAs [249]. In addition, Ψ-containing mRNAs have shown higher stability in cells undergoing stress, suggesting that increased pseudouridylation increases their stability [25–27]. In fact, in vitro-transcribed mRNAs containing pseudouridines that were introduced in mammalian cells or into mouse tissues displayed enhanced stability relative to uridine-containing in vitro-transcribed mRNA [250]. Notably, the majority of pseudouridines in mRNA are regulated in response to environmental signals, but its functional role is still poorly understood [25–27, 220–223].

In tRNAs, pseudouridylation is found at the anticodon stem and loop, at the D stem and in a conserved at the Ψ loop, position 55, all of which stabilize its tertiary structure and are important for codon–anticodon base-pairing [227] (Fig. 4). Novel insights into the role of tRNA pseudouridylation were recently obtained using human embryonic stem cells (hESCs) and haematopoietic stem and progenitor cells (HSPCs) [230]. This work unveiled that similar to m^5^C deposition at the anti-codon and variable loops, the deposition of Ψ at the eighth position of tRNAs by PUS7 regulated the bioproduction of a specific class of 18-nt 5′ tRNA fragments containing a 5′ terminal oligoguanine motif (mTOGs). In addition, the study showed that the presence of Ψ at position 8 of this novel class of small ncRNAs was required for efficient biding to polyadenylate-binding protein 1 (PABPC1), an integral component of the 5′ cap translation initiation complex, resulting in displacement of PABPC1 from capped mRNAS and reprograming translation [230]. Thus, the study showed that RNA modifications, by regulating the biogenesis of a novel class of ncRNAs, they can specialized the translation machinery.

The role of Ψ on other RNAs remains still poorly understood. For instance, TERC is also pseudouridylated [26], where two specific sites of pseudouridylation have been detected, although there is currently no evidence that this modification may be involved in telomerase activity [251]. In miRNA, depletion of PUS10 results in a marked reduction of the expression levels of mature miRNAs and concomitant accumulation of unprocessed pri-miRNAs, but unexpectedly, this process results independent of the catalytic activity of PUS10 [228].

In sum, Ψ deposition may confer different molecular properties to the modified RNAs, which changes their fate or activity. While most recent studies are focused on Ψ modifications in mRNA, yet its role on mRNA is still unclear. Thus, further studies will be required to fully understand the molecular role of Ψ on mRNAs. Ψ is highly abundant on other RNA types such as snRNA, rRNA or tRNA, and whose role has been studied for many years. Yet, recent findings are still revealing novel and unexpected functions, such as the role of Ψ on tRNA fragments, and thus indicating that other functions are still to be discovered.

### Role of pseudouridylated RNAs in cancer

#### Role of DKC1 in cancer

One of the most studied disease linked to defects in pseudouridylation is the X-linked Dyskeratosis Congenita (X-DC), associated to *DKC1* inactivating mutations [252]. Dyskerin modifies mainly rRNAs and snRNAs and it also participates in the active telomerase complex [219]. X-DC is characterized by defects in reticulate skin pigmentation, nail dystrophy, and mucosal leukoplakia, but bone marrow failure is the principal cause of early mortality in X-DC patients [253]. Patients with X-DC are also characterized by having higher risk for cancer development, and expression alterations are associated to cancer too [254]. DKC1 alterations have been found associated to skin cancer [255], breast [256, 257], colon [254, 258, 259], lung [254, 260], prostate [261], head and neck [262, 263], glioma [264], HCCs [265], and specially bone marrow failure syndromes and hematologic malignancies including chronic lymphocytic leukaemia [266, 267] or multiple myeloma [268–270] (Table 3). From the molecular point of view, DKC1 is a nucleolar protein that participates in the stabilization of the telomerase RNA component, necessary for telomerase activity [271, 272], and pseudouridylation of diverse rRNA residues at important ribosome domains for tRNA and mRNA binding, all of which are important functions in highly proliferating cells [273]. Thus, lack of dyskerin activity causes a reduced replicative potential and premature ageing by an impairment of telomerase activity [251] and impediment of ribosome translation of specific mRNAs [274, 275], primarily affecting tissues with rapid cell turnover (Fig. 5d). Mechanistically, it has been shown that low levels of pseudouridylated rRNA downregulates the internal ribosome entry (IRES)-dependent translation of tumour suppressors such as p53 [276], p27 and inhibitors of apoptosis (Bcl-xL, XIAP) [274, 277]. Increased expression of vascular endothelial growth factor has been associated to loss of DKC1 [278], resulting their depletion in high incidence of cancer development. Other recent studies investigating the role of Ψ in rRNA have also supported the emergent hypothesis of the existence of specialized ribosomes in cancer. In liver cancer cells, aberrant expression of snoRNA24, which mediates the pseudouridylation at U609 and U863 positions in 18S rRNA, leads to changes on tRNA selection efficiency, ribosome elongation rate and translation efficiency influencing cancer cell survival [279]. Other example of pseudouridine modifications in rRNA is the reduction of m^1^acp^3^Ψ modification of rRNA found in colorectal carcinoma, which affects the direct interaction of tRNA with ribosomal P site, altering and deregulating translation in cancer cells [280]

**Table 3:** Role of pseudouridylases in cancer. AML: Acute myeloid leukaemia; CLL: Chronic lymphocytic leukaemia; HCC: Hepatocellular Carcinoma

Factor/Enzyme	Cancer type	Regulation	Mechanism	Ref
Writers				
DKC1	Breast cancer	Downregulated	DKC1 downregulation leads to an impairment of hTR stabilization, telomerase activity and proper rRNA pseudouridylation.	[256, 257]
	CLL	Downregulated	Lower telomerase activity and lower expression of sheltering components, which facilitates telomeric damages.	[266]
	Colorectal and lung cancer	Sporadic mutations	Unknown.	[254]
	Colorectal cancer	Upregulated	DKC1 increases the expression of TERC and rRNA pseudouridylation, promoting proliferation.	[258, 259]
	Glioblastoma	Upregulated	DKC1 upregulates the expression of N-cadherin, MMP-2, HIF1A, CDK2 and cyclin E.	[264]
	Head and neck cancer	Upregulated	Unknown.	[262, 263]
	HCC	Upregulated	Unknown.	[265]
	Lung cancer	Upregulated	High levels of TERC, leading an increased aggressiveness and poor prognosis.	[260]
	Multiple myeloma	Genomic mutation	Telomere length and expression levels of small nucleolar and small Cajal body-specific RNAs.	[268–270]
	Prostate cancer	Upregulated	Increased abundance of several H/ACA snoRNAs.	[261]
	Skin cancer	Sporadic mutations	Unknown.	[255]
	Pituitary cancer	Sporadic mutations	Defected translation of specific mRNAs harbouring internal ribosomal entry site (IRES) elements, including the tumour suppressor p27.	[277]
PUS1	Melanoma and breast cancer	No change	Interaction of steroid receptor RNA activator 1 (SRA1) with retinoic acid receptor-γ (RARγ) in melanoma cells and with oestrogen receptor (ER) in breast cancer cell lines.	[282]
PUS7	Myelodysplastic syndromes/ AML	Loss	Reduced bioproduction of tRNA-derived fragments leading to significantly higher protein synthesis.	[230, 285]
PUS10	Prostate cancer	No change	PUS10 is a coactivator of TNF-related apoptosis inducing ligand (TRAIL)-induced apoptosis.	[283]
	Lung cancer	Genomic alterations	Unknown.	[284]

Although there is more evidence that Dyskerin acts as a tumour suppressor [274, 277, 278, 281], in contrast, other studies have indicated an oncogenic role. For example, in breast cancer, decreased levels of *DKC1* expression, rRNA pseudouridylation and telomere length correlate with better prognosis [256]. In glioma, increased expression of DKC1 and pseudouridylation promote glioma cell growth and migration by inducing the upregulated expression of gliomagenesis regulators, although the direct role of increased pseudouridylation of RNAs and gliomagenesis remains unexplored (Fig. 5e) [264].

The data so far show that alterations in DKC1 expression or activity are significantly associated to cancer, however the exact mechanism may be cell, tissue or DKC1 substrate dependent. Further studies will be required to fully explore whether DKC1 has a pro-oncogenic or tumour suppressive role.

#### Role in cancer of RNA-independent pseudouridine synthetases

Despite the significant association of DKC1 activity or expression alterations to cancer, little is known on the role of other pseudouridylases and only few studies have associated alterations on their expression or activity to cancer (Table 3). For example, PUS1 mediates the interaction of steroid receptor RNA activator 1 (SRA1) with retinoic acid receptor-γ (RARγ) in melanoma cells, and with oestrogen receptor in breast cancer cell lines [282]. PUS10 is a mediator of TNF-related apoptosis inducing ligand (TRAIL)-induced apoptosis in prostate cancer cells [283], and genomic alterations of *PUS10* locus are significantly associated with lung cancer risk [284], although it is not clear whether this effects are dependent on pseudouridylase activity. Loss of *PUS7* occurs frequently in myelodysplastic syndromes, haematological clonal disorders characterised by haematopoietic stem cells dysfunction and high risk of transformation to AML [285]. Mechanistically, Guzzi et al. demonstrated that PUS7 depletion in hESC and HSPCs reduced PUS7-mediated pseudouridylation in a special class of tRNA-derived RNA fragments, inducing significantly higher protein synthesis rates leading to dramatic growth and differentiation defects (Fig. 5f) [230]. Thus this work supports the emerging view that ESCs and cancer cells are highly sensitive to perturbations of protein synthesis, and highlights an important role for tRNA fragment in reprograming translation [286]. Additional work using in vivo models and patient-derived cancer cells will be necessary to explore the clinical implications of targeting PUS7 loss or aberrant tRNA fragment bioproduction in cancer.

### Targeting pseudouridine synthetases in cancer

From the clinical point of view, PUSs or Ψ may serve as potential anti-cancer targets and biomarkers. For instance, high amounts of Ψ have been detected in urine of colon, prostate or ovarian cancer patients, plasma of ovarian patients or in salivary metabolites of oral squamous cell carcinoma patients, suggesting to be a potential biomarker in liquid non-invasive biopsies for early cancer diagnoses [261, 287–290]. Yet, while mutations and expression alterations in *DKC1* have been significantly reported in cancer, little has been reported on the status of other Ψ synthetases. With the advent of cancer genome- and transcriptome-wide studies, future computational analyses will reveal the mutational and expression status of all known human Ψ synthetases.

Regarding the design of drugs or screening for small molecules that inhibit PUSs activity, while several studies have attempted to generate or find compounds to diminish DKC1 activity as potential anti-cancer treatments, very little progress has followed [291–293]. Clinical trials were performed in ovarian carcinoma [294], sarcoma [295], colorectal carcinoma [296], acute myelogenous leukaemia [297], breast cancer [298], lung cancer [299], melanoma [300] and other cancers [301] to examine the effectivity as anti-cancer therapy of pyrazofurin, a small molecule inhibitor of the orotodine-5′-monophosphate-decarboxylase (ODCase) which also inhibits DKC1. In all cases pyrazofurin failed to demonstrate efficient anti-cancer activity. However, whether pyrazofurin could be an effective treatment for patients with overexpressed DKC1 was not concluded from those studies, since DKC1 expression levels were not taken into account.

Another potential small molecule inhibitor that is already used as effective anti-cancer agent in the clinic is 5’FU. Treatment with 5’FU is known to improve survival in various cancers [302]. While the mechanism of action for active metabolites of 5’FU has been always attributed to disruption of both DNA and RNA syntheses and DNA damage induction [302], 5’FU was shown to inhibit pseudouridine synthases, due to the substitution of uracil by the analogue 5’FU in RNAs [303, 304]. In those studies, *Samuelsson et al.* demonstrated that the use of fluorinated tRNAs generated specific and stable non-covalent complexes with yeast pseudouridine synthases, thus acting as potent and irreversible inhibitor [304]. Nonetheless, 5’FU is not an universal inhibitor for all pseudouridine synthases, in fact TruB *E. Coli,* and most likely its eukaryotic homologues, cannot form covalent adducts with fluorinated RNAs [303]. Thus, it would be interesting to test the specificity and efficacy of 5’FU in inhibiting mammalian pseudouridine synthases. In addition, it would be important to determine whether part of the associated cytotoxicity of 5’FU is due to an overall decrease of RNA pseudouridylation or to the loss of a particular modified RNA (e.g. rRNA).

More recent studies have used in silico approaches which can predict possible new inhibitors. One study predicted the use of a small molecule inhibitor based on the disruption of the interaction of DKC1 with TERC [293]. The virtual docking-based screen found ten molecules with high affinity values, of which three resulted as potent telomerase inhibitors in a breast cancer cell line. In another study, *Floresta* et al. hypothesized that nucleoside analogues such as the isoxazolidinyl derivative 5′-monophosphate could act as an inhibitor of pseudouridine 5′-monophosphate glycosidases competing with the natural substrate and hampering the glycosidic C–C bond cleavage [292]. They indeed found that the isoxazolidinyl derivative accommodated within the active site of the enzyme with higher ligand efficiency than the natural substrate, leading to the enzyme inhibition in vitro. While we still ignore the tumour growth inhibitory potential and the therapeutic benefits of using those first inhibitors, these studies set the basis to continue with the search for Ψ synthetase inhibitors for cancer treatment.

## Conclusion

RNA modifications have emerged as critical posttranscriptional regulators of gene expression programmes. We start to appreciate the functional networks that the epitranscriptome interacts with, ranging from metabolisms [31] to epigenetics and chromatin remodelling [24, 216] or the immune system [116]. Despite the progress, most studies have focused on the molecular and physiological functions of only one mark, 6-methyladenosine on mRNA, however the epitranscriptome embraces over 170 RNA chemical modifications that decorate coding and ncRNAs, several other posttranscriptional RNA processing events, and RNA binding proteins that may be as well modified as histones in DNA [1]. Thus, association studies of hundreds of other RNA marks on coding but also ncRNAs such as tRNA or rRNA remain to be explored.

To achieve this, we first need to develop system-wide methods and tools for rapid and quantitative detection of RNA modifications. Most of the stablished methods rely on next-generation sequencing and, as such, they are typically blind to nucleotide modifications. Consequently, indirect methods are required that are based on immunoprecipitation techniques using specific antibodies, or enzymatic methods and chemical labelling and unique base-modification properties of RNA pairing [305]. These methodologies have allowed us to catalogue and identify endless modifications with high precision and at nucleotide resolution, yet these strategies have some limitations, reproducibility rate is low due to technical limitations and poor computational methods. For example, antibodies used to recognize modifications such as m^6^A still exhibit non-specific binding and can bind to N^6^,2′-O-dimethyladenosine (m^6^Am) too [59]. In fact, all antibody-related approaches suffer from poorly characterized, and thus unpredictable specificity of the antibody used for enrichment [306]. To overcome these limitations, novel detection methods have been introduced such as DART-seq (deamination adjacent to RNA modification targets), an antibody-free method for detecting m^6^A sites, where the cytidine deaminase APOBEC1 is fused to the m^6^A-binding YTH domain [307]. Yet, this methodology is also limited since it allows to simultaneously map only RNAs bound to YTH domain containing readers. For m^5^C detection, bisulphite-RNA sequencing is the gold standard, yet the reproducibility is low, especially in low abundant and unstable RNA such as mRNAs [16]. The large initial amount of RNA required to compensate for the high losses caused by the treatment, the resistance to C-U conversion from neighbouring modifications or double-stranded sequences, the inability to differentiate from other modifications protecting the C from the conversation such as 5-hydroxymethylation or 3-methylcytosine, and poor computational analysis are the most common found difficulties [16]. Yet, careful assessment of C-U conversion together with development of statistically robust bioinformatic tools that highly refined for data analysis are still generating contradictory results [155, 156]. Regarding the detection of pseudouridine, several labs have developed methodologies that rely only the chemical treatment of RNAs with CMCT, resulting in little overlap of pseudouridine sites on mRNA from the different studies [25, 26, 221]. Thus, despite to the variety of the established techniques, the reality is that there is currently no generic and precise method for mapping and quantifying modifications in RNA. In addition, current methods are complex and lack of single molecule resolution. In this regard, the emerging third-generation sequencing technologies, such as the platforms provided by Oxford Nanopore Technologies (ONT) and Pacific Biosciences (PacBio) have been proposed as a new means to directly detect RNA modifications [308]. RNA modifications can be detected by kinetic changes of reverse transcriptases when encountering a modified nucleotide (PacBio) [309]. Or by current changes as the native RNA molecule is pulled through a membrane pore (ONT) [310]. Although the detection of modifications using ONT direct RNA sequencing is already a reality [311], yet current efforts have not yielded an efficient and accurate RNA modification detection algorithm, largely due to the challenges in the alignment and re-squiggling of RNA current intensities. But emerging alternative base-calling strategies such as *EpiNano* algorithms which identifies m^6^A from RNA reads with an overall accuracy of ~90%, open new avenues to explore additional RNA modifications in the future [312].

The dynamic expression patterns of writer, reader and eraser proteins complicate the identification of the precise functional consequences of aberrant deposition of modifications on RNA metabolism. Thus uncovering the complete repertoire of cellular RNA substrates and the writers, readers, and erasers will unveil how the intricate network of epitranscriptomic events can converge into similar cellular processes and showing how their unbalanced deposition may lead to pathologies. For example, *HIF1A* mRNA is stabilized by m^6^A deposition, while in melanoma cells high levels of HIF1α protein are maintained by modifications at the U34 wobble position of tRNAs [51, 143]. Furthermore, it will be essential to understand the factors or signals that determine the specificity of the RNA modification writers, readers, and erasers and how these proteins are regulated in different cell types. For instance, METTL16 is sensitive to SAM levels and can regulate its synthesis by modifying the SAM synthase gene MAT2A as a feedback loop mechanism [313]. In addition, we need to develop innovative technologies for precise manipulation of the epitranscriptome and functional assays that enables to understand their dynamic mechanisms of action of each modified RNA, since depletion of individual RNA modifier may not be sufficient to comprehensively understand their roles. For example, while the main target for NSUN2 are tRNAs, it still remains unclear whether the phenotypic changes seeing upon *Nsun2* deletion are caused by decrease methylation of tRNAs, or other RNA substrates may as well contribute to the observed phenotype [42, 168, 179, 181, 182].

We start to appreciate the wide range of functional consequences of the aberrant deposition of RNA modifications in human diseases including cancer. For example, aberrant deposition of tRNA modifications, including Ψ and m^5^C, leads to perturbed accumulation of tRNA fragments, a novel class of functional ncRNAs whose role is associated with aberrant protein synthesis rates and reprograming of the translational machinery in tissue and cancer stem cell populations [33, 230]. These findings support the current view that balanced protein synthesis and tight control of mRNA translation is central to cellular processes involved in tumorigenesis [314] and highlights a key role for tRNAs and tRNAs fragments in tumourigenic processes [315]. Yet, our understanding on how specific species of tRNA fragments govern these processes and the clinical implications of their aberrant expression are still very limited. Future studies will be necessary to decipher the molecular bases of the translation reprograming driven by tRNA fragments. Additional work will be necessary to differentiate the contribution of global changes in tRNA pools versus specific population tRNA-derived fragments in the tumour promoting effect of aberrant protein synthesis [315]. More importantly, given their association to cancer progression, their therapeutic potential must be explored exploiting the current advent in miRNA-based therapeutics [316].

Recent advancements in the rapidly evolving field of epitranscriptomics have linked the reprogramming of components of the epitranscriptomic machinery including writers, erasers or readers of the m^6^A, m^5^C or Ψ to cancer. The extensive number of RNA modifications that constitute the epitranscriptome and the reversible nature of epitranscriptomic aberrations hold promise to the emergence of a promising field of epitranscriptomic therapy, which is already making progress with the recent development of effective inhibitors against m^6^A modifying proteins. In the last years, several seminal studies have shed light onto the diagnostic and therapeutic potential of targeting the cancer epitranscriptomic code [3, 33, 317], yet we are far from understanding whether spatio-temporal modifications of the epitranscriptome can drive tumour initiation [318]. In addition, their use as therapeutic agents remains a great challenge due to the lack of consistent and consolidated evidence on the oncogenic or tumour suppressive nature of for example the aberrant deposition of m^6^A. The inconsistent evidence may reflect the precise functional outcome of the RNA modification on each modified RNA type, their crosstalk with other active signalling processes and the dynamic nature of the epitranscriptome. Identifying accurate epitranscriptome biomarkers and defining the oncogenic or tumour suppressive effect of a given aberrant modification within a specific cellular context, cell type, cellular proliferative capacity or tumour microenvironment will guide finding the exact molecular targets to develop selective and effective therapies for a given tumour type.

Though aberrant expression of several methylases and Ψ synthetases has now been described in cancer, it remains unclear whether they could be efficient targets for cancer therapy. Thus, the precise contribution of methylases and Ψ synthetases to tumour initiation, growth, metastasis and resistance need to be further investigated. It remains unclear the RNA target specificity of each enzyme and how specific modified targets can contribute to the malignant phenotype. In addition, little is known on the dynamics of the deposition of these modifications, their erasers and how the binding to their readers influence the RNA metabolism and tumour cell fate. Yet, the availability of the 3D structure of most of these enzymes and the fact that potent and selective inhibitors have been found [52], it is reasonable to expect that inhibition of these enzymes is achievable. These structures can provide the basis for structure-guided drug design which in combination with computational tools can be powerful resources for the development of RNA modifying enzymes inhibitors. Few small molecule inhibitors have been described that can target specifically m^6^A erasers, yet none of them have reached clinical stages [52]. For m^5^C methylases, azacytidine and decitabine (5-aza2′-deoxycytidine), which are cytidine analogues and can inhibit any cytosine-5 methylase, have been approved for clinical use in haematological malignancies [319]. However, their use should be taken carefully due to the lack of specificity of these inhibitors that can inhibit both RNA or DNA cytosine methylases. The validation of these enzymes and their modifications as good pharmacological targets will require the discovery of potent, selective, cell-permeable inhibitors to determine the therapeutic benefit and potential risks associated with inhibiting these enzymes.

Treatment failure in certain settings has been attributed to the presence of sub-populations of cancer stem cells or persister cells which are intrinsically resistant to many therapeutic approaches [320]. Given that m^6^A, m^5^C or Ψ regulate cell survival to stress in many settings and stem cell functions, targeting them represent a very promising opportunity to specifically target these cells populations and reduce chemoresistance and recurrence. Whether other epitranscriptomics changes can lead to drug resistance is not yet understood. This clearly opens the opportunity to explore novel avenues to develop diagnostic tools based on epitranscriptomic signatures that allow for better patient stratification. In addition, combinatorial therapeutic strategies with the potential to re-establish the normal epitranscriptomic landscape or inhibit survival signalling pathways are promising strategies to specifically eliminate cancer stem cells. Combinatorial therapies that target independent pathways may be a better option as the possibilities for the development of tumour drug resistance may be more limited. Understanding the machineries and factors that introduce, remove, or read RNA modifications will allow the development of novel drugs with pharmaceutical value, not only for cancer but other complex human pathologies that have been linked to aberrant deposition of RNA modifications such as diabetes, neurological, immune and mitochondrial-linked disorders [47].

## Data Availability

Supporting data will be available upon request to corresponding author.

## Abbreviations

6PGD: 6-Phosphogluconate dehydrogenase
ADAM19: ADAM metallopeptidase domain 19
AFF4: AF4/FMR2 family member 4
AKT: AKT serine/threonine kinase
ASB2: Ankyrin repeat and SOCS box containing 2
AXL: AXL receptor tyrosine kinase
BCL-2: B cell leukaemia 2
BNIP3: BCL2 interacting protein 3
MET: MET proto-oncogene, receptor tyrosine kinase
CDK2: Cyclin dependent kinase 2
CEBPA: CCAAT enhancer binding protein alpha
CMCT: Carbodiimide(N-Cyclohexyl-N′-(2-morpholinoethyl) carbodiimide metho-ρ-toluenesulfonate
CXCR4: C-X-C motif chemokine receptor 4
E2F1: E2F transcription factor 1
EGFR: Epidermal growth factor receptor
EIF3C: Eukaryotic translation initiation factor 3 subunit C
ELAVL1: ELAV like RNA binding protein 1
ERCC1: ERCC excision repair 1
ERK: Mitogen-activated protein kinase 1
ESR1: Estrogen receptor 1
FOXM1: Forkhead box M1
GLI1: GLI family zinc finger 1
HBXIP: Hepatitis B virus X-interacting protein
HIF1A: Hypoxia inducible factor 1 subunit alpha
HINT2: Histidine triad nucleotide binding protein 2
ITGA6: Integrin subunit alpha 6
ITGB1: Integrin subunit beta 1
JUNB: JunB proto-oncogene, AP-1 transcription factor subunit
KCNK15-AS1: KCNK15 and WISP2 antisense RNA 1
KLF4: Kruppel like factor 4
LATS-2: Arge tumor suppressor kinase 2
LEF1: Lymphoid enhancer binding factor 1
Let-7 g: MicroRNA let-7 g
MALAT1: Metastasis-associated lung adenocarcinoma transcript 1
MEK: Mitogen-activated protein kinase kinase 7
MMP2: Matrix metallopeptidase 2
mTOR: Mechanistic target of rapamycin kinase
MYB: Myeloblastosis oncogene
MYC: Myelocytomatosis oncogene
MZF1: Myeloid zinc finger 1
NANOG: Nanog homeobox
NEAT1: Nuclear paraspeckle assembly transcript 1
NF-κB: Nuclear factor kappa B subunit 1
NMR: Long intergenic non-protein coding RNA 1672
NOP2: NOP2 nucleolar protein
NSUN: NOP2/Sun RNA methyltransferase
P2RX6: Purinergic receptor P2X 6
P53: Tumor protein p53
PDCD1: Programmed cell death 1
PI3K: Phosphatidylinositol 3-kinase,
Pri-miRNA: Primary microRNA
Pre-miRNA: Precursor microRNA
PRMT1: Protein arginine methyltransferase 1
PTEN: Phosphatase and tensin homolog
RARA: Retinoic acid receptor alpha
SETD7: SET domain containing 7, histone lysine methyltransferase
SHH: Sonic hedgehog signaling molecule
SOCS2: Suppressor of cytokine signaling 2
SOX10: SRY-box transcription factor 10
SOX2: Sex determining region Y box 2
SPRED2: Sprouty related EVH1 domain containing 2
TAZ: Tafazzin
TERC: Telomerase RNA component
TET: TET methylcytosine dioxygenase
TNF: Tumor necrosis factor
TNFRSF2: TNF receptor superfamily member
VASH: Vasohibin 1
WAF-1: Cyclin dependent kinase inhibitor 1A
XIAP: X-linked inhibitor of apoptosis
XIST: X inactive specific transcript
YAP: Yes1 associated transcriptional regulator
